# A scoping review of indirect comparison methods and applications using individual patient data

**DOI:** 10.1186/s12874-016-0146-y

**Published:** 2016-04-27

**Authors:** Areti Angeliki Veroniki, Sharon E. Straus, Charlene Soobiah, Meghan J. Elliott, Andrea C. Tricco

**Affiliations:** Li Ka Shing Knowledge Institute, St. Michael’s Hospital, 209 Victoria Street, East Building, Toronto, ON M5B 1T8 Canada; Department of Geriatric Medicine, Faculty of Medicine, University of Toronto, 27 King’s College Circle, Toronto, ON M5S 1A1 Canada; Institute of Health Policy, Management and Evaluation, University of Toronto, Health Sciences Building, 155 College Street, 4th floor, Toronto, ON M5T 3M6 Canada; Epidemiology Division, Dalla Lana School of Public Health, University of Toronto, 155 College Street, 6th floor, Toronto, ON M5T 3M7 Canada

**Keywords:** Network meta-analysis, Individual participant data, Patient-level data, Multiple treatments meta-analysis, Knowledge synthesis, Research methods, Scoping review

## Abstract

**Background:**

Several indirect comparison methods, including network meta-analyses (NMAs), using individual patient data (IPD) have been developed to synthesize evidence from a network of trials. Although IPD indirect comparisons are published with increasing frequency in health care literature, there is no guidance on selecting the appropriate methodology and on reporting the methods and results.

**Methods:**

In this paper we examine the methods and reporting of indirect comparison methods using IPD. We searched MEDLINE, Embase, the Cochrane Library, and CINAHL from inception until October 2014. We included published and unpublished studies reporting a method, application, or review of indirect comparisons using IPD and at least three interventions.

**Results:**

We identified 37 papers, including a total of 33 empirical networks. Of these, only 9 (27 %) IPD-NMAs reported the existence of a study protocol, whereas 3 (9 %) studies mentioned that protocols existed without providing a reference. The 33 empirical networks included 24 (73 %) IPD-NMAs and 9 (27 %) matching adjusted indirect comparisons (MAICs). Of the 21 (64 %) networks with at least one closed loop, 19 (90 %) were IPD-NMAs, 13 (68 %) of which evaluated the prerequisite consistency assumption, and only 5 (38 %) of the 13 IPD-NMAs used statistical approaches. The median number of trials included per network was 10 (IQR 4–19) (IPD-NMA: 15 [IQR 8–20]; MAIC: 2 [IQR 3–5]), and the median number of IPD trials included in a network was 3 (IQR 1–9) (IPD-NMA: 6 [IQR 2–11]; MAIC: 2 [IQR 1–2]). Half of the networks (17; 52 %) applied Bayesian hierarchical models (14 one-stage, 1 two-stage, 1 used IPD as an informative prior, 1 unclear-stage), including either IPD alone or with aggregated data (AD). Models for dichotomous and continuous outcomes were available (IPD alone or combined with AD), as were models for time-to-event data (IPD combined with AD).

**Conclusions:**

One in three indirect comparison methods modeling IPD adjusted results from different trials to estimate effects as if they had come from the same, randomized, population. Key methodological and reporting elements (e.g., evaluation of consistency, existence of study protocol) were often missing from an indirect comparison paper.

**Electronic supplementary material:**

The online version of this article (doi:10.1186/s12874-016-0146-y) contains supplementary material, which is available to authorized users.

## Background

Systematic reviews and meta-analyses using individual patient data (IPD) aim to obtain, verify, and synthesize original research data for each participant from all studies that compare the same two treatments to address a specified clinical question. Although IPD meta-analyses may be more time consuming and expensive than conventional meta-analyses using aggregated data, they are considered the gold standard approach for systematic reviews of interventions and are being published with increasing frequency [1, 2]. They can improve clinical practice guidelines [3] because they offer advantages over conventional meta-analyses with respect to data quality and the type of analyses that can be conducted. For example, in contrast to aggregated data, the use of IPD allows investigation of patient-level moderators, intention-to-treat analysis (when data are available for all patients in randomized studies), and application of appropriate multiple imputation techniques to overcome issues related to missing data.

Network meta-analysis (NMA) allows the simultaneous comparison of many relevant interventions, and there has been an exponential increase in the number of NMAs published in recent years [4]. Although NMA is commonly performed with aggregated data, the inclusion of IPD can increase confidence in the results [5, 6], identify interactions that are otherwise undetectable [1, 7–9], and reduce variation in treatment effects both between studies within pairwise comparisons (heterogeneity) and between pairwise comparisons (inconsistency) by adjusting trial results for factors that may cause this variation [6]. The use of IPD may also allow estimation of subgroup effects, which in turn allows tailoring of results to patient characteristics. Several investigators have recognized that the use of IPD in NMAs may generate the most trustworthy evidence to inform clinical decision making, and hence they have been developing statistical methods to enhance IPD-NMAs [5, 6, 10, 11]. The objective of this study is to conduct a comprehensive scoping review of the methods used to perform indirect comparisons with IPD or IPD combined with aggregated data. We also aim to review applications of indirect comparisons with IPD and summarize network, methods and reporting characteristics.

## Methods

This review was guided by the research questions: “What are the existing methodologies available to apply an IPD-NMA or an indirect comparison using IPD?" and "What are the characteristics of the empirical networks that include IPD (e.g., number of trials, patients, and treatments)?”. A scoping review was applied for this study based on the framework outlined by Arksey and O’Malley [12] and using the Joanna Briggs Institute methods manual [13]. We described the methods in detail in our protocol publication [14].

### Identifying relevant studies: data sources and search strategy

We searched MEDLINE, Embase, the Cochrane Library, and CINAHL from inception until the end of October 2014. No limits were placed on date of publication, language, population, intervention, or outcome. The search was carried out by an experienced librarian (Ms Becky Skidmore), and a second librarian (Ms Heather MacDonald) peer-reviewed the MEDLINE electronic search strategy (see Additional file 1: Appendix 1) using the Peer Review of Electronic Search Strategies (PRESS) checklist [15]. Modified search strategies for remaining databases are available upon request from the authors. Grey literature sources (Google, Agency for Healthcare Research and Quality, Canadian Medical Libraries List, Medical Research Council, and National Health Service) were searched, and references from included studies were scanned.

### Eligibility criteria

We included published papers, protocols, and abstracts, as well as unpublished studies, that reported on a method, application, or review of IPD indirect comparison methods involving studies of any design. Eligible were application studies that compared the clinical effectiveness or safety of three or more interventions and applied any type of indirect comparison, including adjusted indirect comparison, unadjusted indirect comparison, matching adjusted indirect comparison (MAIC), simulated treatment comparison (STC), mixed comparison, and NMA. Studies including narrative comparisons were excluded.

Several approaches have been suggested to conduct an indirect comparison using IPD only or in combination with aggregated data. The different types of IPD indirect comparison methods identified in this scoping review are outlined in Table 1. The adjusted indirect comparison, mixed comparison, and NMA approaches modeling IPD can be categorized as one-stage and two-stage approaches. In one-stage methods, the IPD from all eligible studies are analyzed within the same (usually linear) model simultaneously, accounting for clustering of participants within each study. Two-stage methods are used to reduce IPD to aggregated data and then synthesize the aggregated data from each study using an adjusted indirect comparison, mixed comparison, or NMA model [16].

**Table 1:** Individual patient data indirect comparison methods

• *Adjusted indirect comparison*: The method derives an indirect estimate for the relative effectiveness or safety of two different treatments adjusted by comparing the results of their direct comparisons (i.e. pairwise meta-analyses) with a common comparator treatment [68]. Consider, for example, a tree-shaped triangular network composed by some IPD (or IPD and aggregated data) studies comparing treatment A against treatment B, and some studies comparing treatment A against treatment C. The method uses the summary treatment effect estimates derived by a pairwise meta-analysis (which can be a one-stage or two-stage approach) for studies Avs.B and for studies Avs.C. • *Matching adjusted indirect comparison (MAIC)*: The method estimates an indirect comparison of the treatments of interest [48]. Consider the tree-shaped triangular network ABC composed by some IPD studies comparing Avs.B treatments and some aggregated data studies comparing Avs.C treatments. The method uses the information from the IPD trials on one treatment arm (B) and the information from the aggregated data trials on the other treatment of interest (C). The patient characteristics from the IPD trials on treatment B are then matched to the ones of the aggregated data trials on treatment C using an approach similar to propensity score weighting. Specifically, the patient baseline characteristics in IPD trials and treatment B are reweighted so that the weighted average of the patient characteristics matches the characteristics of the population in treatment C of the aggregated data trials. The weights are modeled as a linear combination of all reported baseline characteristics. After matching the baseline characteristics between the two groups, the treatment outcomes are compared across the trial populations using the adjusted mean for treatment B and observed mean for treatment C. • *Simulated treatment comparison (STC)*: A similar approach to the MAIC is the STC, which uses a different process to adjust for population characteristics [49]. Considering the same tree-shaped ABC network, the STC method estimates the treatment response in B using information from the IPD trials and a predictive regression model with patient-level characteristic covariates. Then a statistical calibration of the trial(s) with IPD is performed to match the characteristics with aggregated data trials and treatment C. Trial data are simulated for treatment B based on the statistical calibration. The adjusted mean for treatment B is compared with the observed mean for treatment C. The MAIC and STC methods may be particularly useful when there is insufficient data from head-to-head comparison trials, and when there is insufficient data to apply an adjusted indirect comparison (e.g., disconnected network of trials) [50]. • *Network meta-analysis (NMA) approaches*: When both direct and indirect evidence (IPD or in combination with aggregated data or aggregated data only) are available for the same comparison (e.g., Bvs.C), then these may be combined in a mixed effect size using the mixed comparison method [69]. The mixed comparison estimate is a weighted average of the meta-analytic effect estimate Bvs.C and the adjusted indirect comparison for Bvs.C. A suggested approach for combining direct and indirect evidence using the mixed comparison method is the inverse variance method with weights the inverse of the variance of the estimated effects. An approach to simultaneously compare multiple treatments in a single analysis is by using a meta-regression model [70]. Each study-specific treatment effect is expressed as a linear function of the basic parameters, which is a set of comparisons of the treatments in the network versus the reference treatment. Assuming A is the reference treatment then Avs.B and Avs.C are the basic parameters for the ABC network. This approach uses the different treatment comparisons as covariates in the meta-regression model. In particular, it uses dummy variables for the basic parameters to define the basic contrasts Avs.B and Avs.C, omits the intercept, and specifies the covariate values so that consistency between direct and indirect evidence holds. An alternative way to apply a NMA is by using hierarchical models [71]. Studies have shown that the majority of NMA applications have been carried out in a Bayesian setting using hierarchical models [4, 10, 42].	

### Study selection and data abstraction

Following a calibration exercise, two reviewers (AAV and CS or MJE) independently screened each title and abstract of the literature search results (level 1) and the full-text of potentially relevant articles (level 2) using Synthesi.SR [17]. Conflicts were resolved by discussion. The final inter-rater agreement (across levels 1 and 2) between reviewers was 85 %. The same process was followed for data extraction. When multiple publications were identified for the same study, we abstracted data from the most recent study (when the literature search differed across studies) and considered the remaining publications as companion reports, which were used for supplementary material only. Details on the data abstraction process can be found in Additional file 1: Appendix 2.

### Synthesis

Quantitative data from the retrieved networks with IPD (e.g., number of patients, studies, and treatments in the network) were summarized in terms of medians and interquartile ranges (IQRs), and categorical data (e.g., effect measures, outcome data type, reference treatment type) by frequencies and percentages. We compared continuous network characteristics between different methods using the Wilcoxon-Mann-Whitney test. All tests were two-sided with a significance level of 0.05.

## Results

**Fig. 1:**
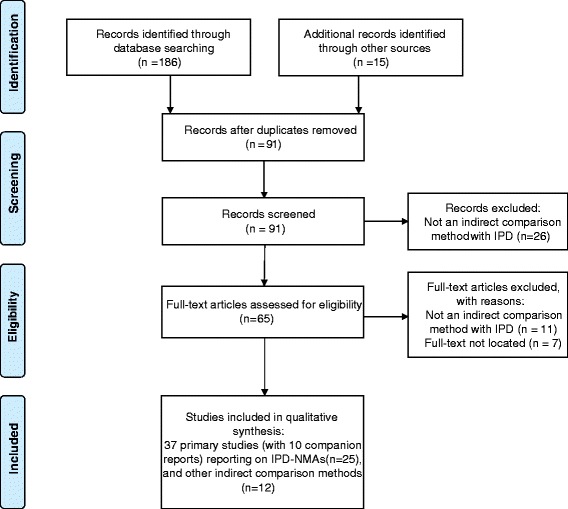
PRISMA flow chart for study selection. IPD-NMA = individual patient data network meta-analysis

The literature search yielded 201 potentially relevant citations, of which 91 unique citations met the eligibility criteria based on title and abstract. Following review of the corresponding full-text articles, 37 papers were eligible for this review and included, along with 10 companion reports (Fig. 1). All excluded citations and reasons for exclusion are available in Additional file 1: Appendix 3.

### General characteristics of identified networks

**Fig. 2:**
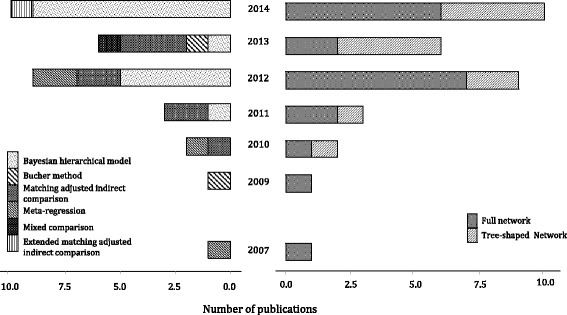
Bar plot of the indirect methods using individual patient data (IPD) by year, method, and type of network. The frequencies of the identified methods (*n* = 33) were 17 (52 %) Bayesian hierarchical models†, 2 (6 %) Bucher methods‡, 8 (24 %) matching adjusted indirect comparisons (MAIC)^#^, 1 (3 %) extended MAIC^#^, 4 (12 %) meta-regression models*, 1 (3 %) mixed comparison**. †Bayesian hierarchical models are multi-level models presented as a generalization of regression methods. Different levels account for the variation in patients between and within studies which form the hierarchical model. Network meta-analyses conducted in a Bayesian framework express the observed treatment effects via their ‘true’ underlying treatment effects. ‡The Bucher method (or adjusted indirect comparison) is the statistical approach to derive an indirect treatment effect estimate for two competing treatments that have been compared with a common intervention [68]. ^#^Matching-adjusted indirect comparisons are indirect comparisons that use IPD from the active treatment trial(s) and aggregate data (AD) from the comparator treatment trial(s). The patient characteristics from the IPD trial(s) are weighted *a priori* and matched with the characteristics of the population in the AD trial(s) so that the baseline characteristics are similar between the two treatment groups. A recent extension of the method accounts for differences in endpoint definitions and missing data [46]. *A linear (or meta-regression) model with dummy variables reflecting the basic parameters (comparisons of all treatments vs. a common comparator), and with regression coefficients the NMA treatment effect estimates [69]. Under the consistency assumption, all treatment comparisons are written as functions of the basic parameters. **A mixed comparison between two treatments is the weighted average of direct and indirect estimates for the same treatment comparison, with weights the inverse of the variance of the estimated effects [69]

We identified 23 (62 %) application articles [18–40], 11 (30 %) methodological articles [6, 41–49], 2 (5 %) reviews of methods [50, 51], and 1 (3 %) protocol [52] for an application article that has not yet been published (Additional file 1: Appendix 4). The number of studies with indirect comparison methods using IPD has increased steeply since 2007 (Fig. 2). The IPD indirect comparison methods were published in a wide variety of journals, and most of the networks (17; 46 %) were industry-sponsored. Further details can be found in Additional file 1: Appendix 5.

### Characteristics of identified methodologies

#### Summary of indirect comparison methodologies using IPD

**Fig. 3:**
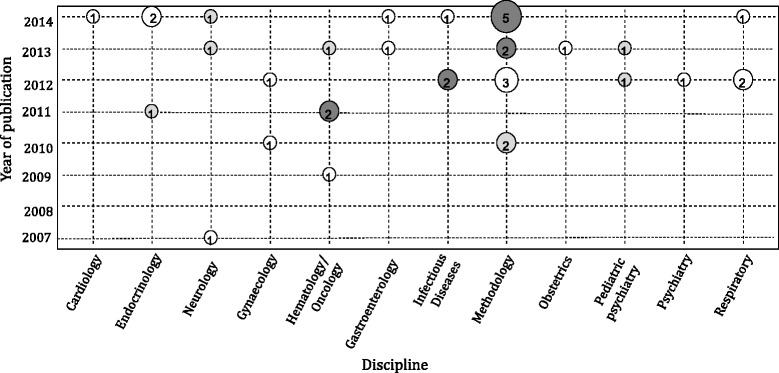
Bubble plot of indirect methods using individual patient data by year of publication and discipline. The size of each bubble is proportional to the number of studies published in the corresponding year and discipline. Light grey bubbles represent publications using the matching adjusted indirect comparison (MAIC) and simulated treatment comparison (STC) methods, white bubbles represent publications using an individual patient data network meta-analysis (IPD-NMA) method, and dark grey bubbles represent publications using both IPD-NMAs and MAIC/STC methods

A variety of indirect comparison methods using IPD were identified (Table 2). Twenty-four IPD-NMA (73 %) and 9 MAIC (27 %) approaches were applied in total in the empirical studies. The first IPD-NMA study, published in 2007, applied a meta-regression model for time-to-event data [19]. About half of the networks (17; 52 %) applied a Bayesian hierarchical model, whereas the second most frequently used method was the MAIC approach (8; 24 %) (Fig. 3).

**Table 2:** Properties of methods to derive indirect and network meta-analysis estimates using individual patient data

	Adjusted indirect comparison (or Bucher method)	Mixed comparison	Meta-regression model	Bayesian hierarchical NMA model	MAIC [48]	STC [49]
No. of empirical studies applying method (*n* = 33)	2 (6 %) [20, 52]	1 (3 %) [28]	4 (12 %) [19, 21, 25, 26]	17 (52 %) [6, 10, 18, 22–24, 27, 29–33, 42–45, 51]	8 (24 %) MAICs [34–38, 48, 57] and 1 (3 %) extended MAIC [46]	0 (0 %)
*Properties*						
1-stage or 2-stage process	2-stage	2-stage	Both can be applied	Both can be applied	NA	NA
Format of data	IPD+AD/IPD only	IPD+AD/IPD only	IPD+AD/IPD only	IPD+AD/IPD only	IPD+AD	IPD+AD
Avoids selective use of indirect evidence from a network of trials	No	No	Yes	Yes	No	No
Can compare >2 treatments at a time for efficacy/safety	No	No	Yes	Yes	No	No
Preserves within-trial randomization	Yes	Yes	Yes	Yes	No	No
Study-specific true treatment effects can be assumed as fixed or random with common mean effect for each pairwise comparison	Yes	Yes	Yes	Yes	No	No
May account for potential clinical and methodological differences across trials	Yes	Yes	Yes	Yes	No	No
Does not require assessment for transitivity assumption	No	No	No	No	Yes	Yes
Mean treatment effects expressed via consistency equations	No	Yes	Yes	Yes	No	No
Can rank all competing treatments for same condition	No	No	Yes	Yes	No	No
Enables adjustment for predefined set of patient characteristics	No	No	Yes	Yes	Yes	Yes
Can be applied even in disconnected network of trials	No	No	No	No	Yes	Yes

*AD* aggregated data, *IPD* individual patient data, *MAIC* matching adjusted indirect comparison, *NA* not applicable, *NMA* network meta-analysis, *STC* simulated treatment comparison

Most IPD-NMAs involved one- or two-stage approaches (see Additional file 1: Appendix 4 and Additional file 2). Several one-stage Bayesian hierarchical models were discussed across the methodological papers, including either IPD alone [6, 41–43] or a mixture of IPD and aggregated data [41, 42, 44, 45] (see Table 3). For IPD alone, three studies [6, 10, 41] presented models for dichotomous outcome data using the odds ratio, and a fourth study [43] proposed a model for multiple continuous outcomes using the mean difference. For combining IPD with aggregated data, three studies [41, 42] presented models for dichotomous outcome data using the odds ratio, a fourth study [44] proposed a model for time-to-event data using the hazard ratio, and a fifth study [45] suggested a model for continuous data using the mean difference. All of the aforementioned models were developed to model randomized clinical trials (RCTs), apart from the models suggested by Saramago and colleagues [10], which can combine cluster- and patient-randomized trials, and the approach proposed by Thom and colleagues [45], which models RCTs and single-arm observational trials.

**Table 3:** Bayesian hierarchical IPD-NMA models described in the identified methodological articles

Model	1-stage or 2-stage process	Format of data	Study design	Type of data	Effect size	Assumptions for treatment by covariate interactions
Donegan et al. [6]	1-stage	IPD only	RCTs	Dichotomous	Odds ratio	No interactions; exchangeable treatment by covariate interactions; common treatment by covariate interactions
Donegan et al. [42]	1-stage	IPD+AD and IPD only	RCTs	Dichotomous	Odds ratio	No interactions; independent treatment by covariate interactions; exchangeable treatment by covariate interactions; common treatment by covariate interactions
Hong et al. [43]	1-stage	IPD only	RCTs	Continuous	Mean difference	Exchangeable treatment by covariate interactions
Jansen [41]	1-stage	IPD+AD and IPD only	RCTs	Dichotomous	Odds ratio	Exchangeable treatment by covariate interactions; common treatment by covariate interactions
Saramago et al. [10]	1-stage	IPD+AD and IPD only	Cluster and individual allocation trials	Dichotomous	Odds ratio	Independent treatment by covariate interactions; exchangeable treatment by covariate interactions; common treatment by covariate interactions
Saramago et al. [44]	1-stage	IPD+AD	RCTs	Time-to-event	Hazard ratio	Common treatment by covariate interactions
Thom et al. [45]	1-stage	IPD+AD	RCTs and single-arm observational studies	Continuous	Mean difference	Independent treatment by covariate interactions

*AD* aggregated data, *IPD* individual patient data, *IPD-NMA* individual patient data network meta-analysis, *RCT* randomized controlled trial

The majority (15; 63 %) of the 24 empirical IPD-NMAs used a one-stage analysis; two-stage analysis was the second most frequent method (7, 29 %), one study (4 %) used IPD as an informative prior [32], and one study (4 %) [33] was unclear about the analysis format. Among the 33 networks, 16 (52 %) implemented indirect comparison methods modeling IPD in Bayesian statistics software (JAGS [1; 3 %] [53] OpenBUGS [2; 6 %] [54] WinBUGS [14; 43 %] [55] (Table 4). Of the total 37 papers, only three (8 %) IPD-NMAs [10, 44, 45] provided their code in the manuscript, whereas one (3 %) reported that the code is available upon request [31]. Of the 24 empirical IPD-NMAs, 9 (38 %) used IPD only, 13 (54 %) used a mixture of IPD and aggregated data, and two (8 %) applied a combination of methods using both IPD alone and a mixture of IPD and aggregated data. The data format used in all MAICs was a mixture of IPD and aggregated data. The design of the studies included in all of the empirical networks was an RCT, except for in three studies (9 %), which included non-randomized data [10, 31, 45]. The reasons for the choice between IPD or their combination with aggregated data included the following: (not) having access to IPD, not contacting authors outside the collaborative research group, to use IPD as a prior distribution in the analysis, to assess the benefits of acquiring IPD for a subset of trials, to compare IPD-NMA models with aggregated NMA models, and to apply a MAIC (Additional file 2).

**Table 4:** Methodological characteristics of identified empirical networks, including unpublished data provided by study authors. Figures are no. (%) of studies

Characteristic	IPD-NMA studies^a^	MAIC studies^a^	Total^a^
Design of studies included in analyses			
RCTs	21 (70)	9 (30)	30 (91)
RCTs + observational	1 (100)	0 (0)	1 (3)
RCTs + quasi-RCTs	1 (100)	0 (0)	1 (3)
RCTs, non-RCTs, CBA	1 (100)	0 (0)	1 (3)
Total	24 (73)	9 (27)	33 (100)
Fixed- or random-effects model			
Random-effects model	10 (100)	0 (0)	10 (30)
Fixed-effect model	7 (100)	0 (0)	7 (21)
Fixed- and random-effects models	5 (100)	0 (0)	5 (15)
Not reported/not applicable	2 (18)	9 (82)	11 (33)
Total	24 (73)	9 (27)	33 (100)
Between-study variance estimator/prior			
Non-informative prior	10 (100)	0 (0)	10 (67)
Informative prior	1 (100)	0 (0)	1 (7)
Minimally informative prior	1 (100)	0 (0)	1 (7)
DL [72]	1 (100)	0 (0)	1 (7)
REML [73]	1 (100)	0 (0)	1 (7)
Not reported	1 (100)	0 (0)	1 (7)
Total	15 (100)	0 (0)	15 (100)
Methods used to compare different models			
DIC [74]	10 (100)	0 (0)	10 (30)
Statistical significance of regression coefficients and between-study variance	3 (100)	0 (0)	3 (9)
DIC and residual deviance [74]	2 (100)	0 (0)	2 (6)
Comparison of point estimates and their CIs	0 (0)	2 (100)	2 (6)
AIC and Hosmer–Lemeshow [75, 76]	0 (0)	1 (100)	1 (3)
DIC and AIC [74, 75]	1 (100)	0 (0)	1 (3)
Not applicable	8 (57)	6 (43)	14 (42)
Total	24 (73)	9 (27)	33 (100)
Statistical techniques used for missing participant data			
LOCF	2 (67)	1 (33)	3 (9)
MCMC multiple imputations	2 (100)	0 (0)	2 (6)
ACA	1 (100)	0 (0)	1 (3)
LOCF and ACA	0 (0)	1 (100)	1 (3)
Not reported/unclear	19 (73)	7 (27)	26 (79)
Total	24 (73)	9 (27)	33 (100)
Methods used to rank treatment effectiveness/safety			
Probability of being the best	11 (100)	0 (0)	11 (33)
Not reported/not applicable	13 (59)	9 (41)	22 (67)
Total	24 (73)	9 (27)	33 (100)
Assessment of consistency assumption			
Yes	13 (100)	0 (0)	13 (39)
No/unclear	6 (75)	2 (25)	8 (24)
Not applicable	5 (42)	7 (58)	12 (36)
Total	24 (73)	9 (27)	33 (100)
Methods used to assess consistency assumption			
Informal approaches^b^	8 (100)	0 (0)	8 (62)
Loop-specific approach [68, 77]	1 (100)	0 (0)	1 (8)
Loop-specific approach and back-calculation [68, 77, 78]	1 (100)	0 (0)	1 (8)
Lu and Ades [79]	1 (100)	0 (0)	1 (8)
Lumley [70]	1 (100)	0 (0)	1 (8)
Node-splitting [78]	1 (100)	0 (0)	1 (8)
Total	13 (100)	0 (0)	13 (100)
Inclusion of different treatment doses			
No	18 (75)	6 (25)	24 (73)
Yes	6 (67)	3 (33)	9 (27)
Total	24 (73)	9 (27)	33 (100)
Approaches used to account for different treatment doses			
Lumping doses	4 (80)	1 (20)	5 (56)
Splitting doses	2 (50)	2 (50)	4 (44)
Total	6 (67)	3 (33)	9 (100)
Software			
WinBUGS [55]	7 (100)	0 (0)	7 (21)
SAS [80]	2 (33)	4 (67)	6 (18)
WinBUGS and R [55, 81]	5 (100)	0 (0)	5 (15)
OpenBUGS [54]	2 (100)	0 (0)	2 (6)
WinBUGS and Stata [55, 82]	2 (100)	0 (0)	2 (6)
JAGS and R [53, 81]	1 (100)	0 (0)	1 (3)
Stata [82]	1 (100)	0 (0)	1 (3)
Not reported	4 (44)	5 (56)	9 (27)
Total	24 (73)	9 (27)	33 (100)

*ACA* available case analysis, *AIC* Akaike information criterion, *CBA* controlled before-and-after, *CI* confidence interval, *DIC* deviance information criterion, *DL* DerSimonian and Laird, *IPD-NMA* individual patient data network meta-analysis, *LOCF* last observation carried forward, *MAIC* matching adjusted indirect comparison, *MCMC* Markov chain Monte Carlo, *RCT* randomized clinical trial, *REML* restricted maximum likelihood

^a^Percentages were calculated across the row for IPD-NMA and MAIC/STC, but down the column for the “Total” column. Total number of included studies *n* = 37. Total number of empirical networks *n* = 33. Please note that the empirical networks include 8 methodological and 1 review papers

^b^Informal approaches are comparison of NMA results with results previously published, comparison of NMA results with pairwise meta-analysis results, comparison of IPD-NMA with meta-regression IPD-NMA results, comparison of IPD-NMA with aggregated data NMA results

#### Key methodological components of indirect comparison methods with IPD

Of the 22 empirical IPD-NMAs that reported which model was selected among fixed and random-effects, 10 (45 %) employed a random-effects model, 7 (32 %) applied a fixed-effect model, and 5 (23 %) used both approaches. All but two of the Bayesian random-effects IPD-NMA models [10, 32] used a non-informative prior for the between-study variance parameter. Many networks applied various modeling approaches, which were most frequently compared using the deviance information criterion (13; 40 %). The rank order effectiveness or safety of treatments per network was assessed in 11 (33 %) empirical studies using the probability of being the best. Several authors identified differences in the results, when both IPD methods and aggregated data approaches were applied, such as differences in the consistency evaluation, precision in treatment effects, and significance of treatment effect modifier (Additional file 2).

The majority (26; 79 %) of the 33 empirical studies did not report whether an approach had been applied to handle missing data. The approach most commonly applied to follow the intention-to-treat principle in the identified indirect comparison methods was the last observation carried forward (4; 12 %), where missing values are replaced with the last observed measurement. Thirteen (68 %) of the 19 full IPD-NMAs assessed inconsistency, but only 5 (38 %) of these used statistical approaches for this evaluation. One of the full networks was composed of one closed loop of multi-arm studies, and consistency could not be evaluated because of inherent correlations [27]. Of the 13 IPD-NMAs that assessed the consistency assumption, 5 (38 %) detected inconsistency in their network and used IPD to adjust for differences in effect modifiers across treatment comparisons. Among the nine networks that included different treatment doses, the relationship between treatment and dose was ignored either by lumping (5; 56 %) or splitting (4; 44 %) the doses as if they were different treatments.

#### Methods used to report results in the identified networks

The methods used to report the summary estimates from the analyses varied across the papers. Almost half of the empirical studies (15; 45 %) included a network diagram in the results section or in supplementary material. Tables (14; 42 %) and forest plots (27; 82 %) were the most common methods of reporting the results of indirect comparison methods (Additional file 1: Appendix 6).

### Characteristics of empirical studies

#### Protocol and rationale for using IPD

The 33 studies with empirical indirect comparison methods using IPD, included 23 application articles [18–40], 8 methodological articles with empirical examples [6, 10, 42–46, 48], 1 review [51], and 1 protocol [52] (Additional file 1: Appendix 6). Of these 33 studies, 9 (27 %) IPD-NMAs reported the existence of a study protocol; an additional 3 (9 %) studies (two IPD-NMAs and one MAIC) mentioned that protocols existed [20, 33, 44], but references were not provided, and we were unable to locate them. None of the eight methodological articles cited a study protocol, but 4 of them provided a reference of the original publication of the empirical dataset, which cited a protocol. Around 3 to 4 years were required to publish the final IPD review after the protocol was published (Additional file 2). We identified 22 (67 %) studies in which investigators had access to IPD through a collaborative research group, whereas 9 (27 %) systematic reviews used several methods to contact the original authors and collect IPD. Six studies reported the proportion of contacted authors who provided IPD, and the median proportion of studies that obtained IPD was 68 % (IQR 58–78 %). No IPD review reported reasons for any non-located IPD studies. Our response rate to requests for additional information for 29 papers was 82 % (14/17 authors; some authors were contacted for more than one paper).

Many of the papers reported the rationale for using IPD instead of aggregated data (26; 79 %); these reasons included adjusting for potential confounding factors [4, 6, 21, 23, 29, 30, 32, 34, 42, 48, 56], exploring reasons for heterogeneity and/or inconsistency [6, 10, 20, 23, 31, 42], increasing power to detect treatment effect modifiers [10, 19, 45], overcoming bias (e.g., aggregation bias) [10, 43], producing more precise estimates of treatment effect (even in the absence of treatment-by-covariate interactions) [19, 44], adjusting for differences in patient-level characteristics even when a small number of studies (<10) was available [35, 37, 10], increasing power due to rare events [18], and matching differences in baseline characteristics [35–38, 57]. One of the identified simulation studies evaluated the advantages of including IPD in NMA [5]. In that study, Jansen [5] evaluated the performance of tree-shaped triangular IPD-NMAs modeling a combination of IPD and aggregated data compared with NMAs using aggregated data and showed that an IPD-NMA can considerably reduce bias and increase precision of treatment effect estimates when there is an imbalance in patient-level treatment effect modifiers across comparisons.

#### Primary outcome and competing treatments

The primary outcome was an effectiveness outcome in 31 (94 %) studies and was categorized as objective in 26 (79 %) networks. The median number of outcomes assessed in the eligible networks was one (IQR 1-3) (Additional file 1: Appendix 4 and Appendix 6). About half of the networks (17; 52 %) reported a dichotomous primary outcome, and nine (27 %) included a continuous primary outcome (see Additional file 1: Appendix 6). The empirical networks evaluated a wide range of interventions, pharmacological versus placebo or control being the most common type of intervention comparison (17; 52 %). The median number of participants in the empirical networks was 899 (IQR 310–1735) (for IPD-NMAs, 1342 [IQR 493–2567]; for MAICs, 329 [IQR 221–601]; *P* = 0.024).

#### Size and geometry of the identified networks

We identified 33 empirical networks: 21 (64 %) full networks and 12 (36 %) tree-shaped networks. In Additional file 1: Appendix 7 and Appendix 8 we present the distribution of trials, treatment groups, and patients for each network, shown separately for IPD-NMA and MAIC approaches. The median number of interventions assessed per network was 5 (IQR 3–6) (for IPD-NMAs, 6 [IQR 5–7]; for MAICs, 3 [IQR 3–4]; *P* = 0.003), and the median number of closed loops in full networks was 1 (IQR 0–4) (for IPD-NMAs, 2 [IQR 1–5]; for MAICs, 0 [IQR 0-0]; *P* = 0.002). Most IPD-NMAs (19; 79 %) were applied to full networks (including 13 Bayesian hierarchical models, four meta-regression models, one adjusted indirect comparison, one mixed comparison), whereas most MAIC (7; 78 %) were used for tree-shaped networks.

The median number of trials included per network was 10 (IQR 4–19) (for IPD-NMAs, 15 [IQR 8–20]; for MAICs, 2 [IQR 3–5]; *P* <0.001), and the median number of IPD trials included in a network was 3 (IQR 1–9) (for IPD-NMAs, 6 [IQR 2–11]; for MAICs, 2 [IQR 1–2]; *P* = 0.007). Full networks had a median number of multi-arm studies of 0 [IQR 0–2] (for IPD-NMAs, 0 [IQR 0–3]; for MAICs, 0 [IQR 0-0]; *P* = 0.251). The median number of patients in a network was 3874 (IQR 1162–9830) (for IPD-NMAs, 5310 [IQR 3290–14750]; for MAICs, 997 [IQR 520–1264]; *P* <0.001), and the median number of patients in IPD trials was 1790 (IQR 599–5110) (for IPD-NMAs, 3848 [IQR 1444–5643]; for MAICs, 541 [IQR 350–625]; *P* = 0.007). No application papers using the STC method were identified.

## Discussion

### Recommendations to authors

This study is the first scoping review to provide a comprehensive overview of the methods for completing indirect comparison analyses using IPD. It also describes the methodological and reporting characteristics of empirical networks in healthcare, which will help not only in the design of future simulation studies, but also in refining the preferred reporting items for systematic reviews and meta-analyses (PRISMA) using IPD [58] and developing the PRISMA for IPD-NMAs. This review showed that essential methodological and reporting items suggested to be included by PRISMA-IPD [58] and PRISMA-NMA [59], such as evaluation of the consistency assumption, existence of a study protocol, and methods used to request, collect, and manage IPD, were poorly reported in IPD indirect comparisons. An IPD indirect comparison review should be clearly reported in line with the International Society for Pharmacoeconomics and Outcomes Research (ISPOR), PRISMA-IPD and PRISMA-NMA tools [58–60]. However, given that these guidelines are not specific to IPD indirect comparison methods, we outline some additional information that we suggest be reported in IPD indirect comparisons to improve transparency in Table 5 [58–60]. For example, the rationale for the choice of IPD indirect comparison method should be provided, since different approaches are associated with different properties, and hence they may lead to different and potentially conflicting results.

**Table 5:** Suggested information to report in an individual patient data indirect comparison to supplement ISPOR, PRISMA-IPD and PRISMA-NMA

• Rationale for the IPD indirect comparison method selected. • Timelines to obtain, clean, and analyze data. • Process to identify IPD studies, and if authors were contacted, which methods were used to contact them, how many reminders were sent, and who requested the IPD. • Whether the obtained IPD were anonymized. • Mechanism and strategy for storage of IPD. • Whether IPD were requested from all studies or just a subset of studies; report reasons for all missing IPD studies. • Potential legal agreements to access IPD and difficulties encountered due to data protection and intellectual property issues. • Estimator or prior for the between-study variance and rationale for this selection, when a random-effects model is applied. • Software in which the indirect comparison was performed and the statistical code used.	

### Comparison with existing evidence

The IPD indirect comparisons are only a minority of the aggregated data indirect comparisons, which is also true for IPD meta-analyses compared to aggregated data meta-analyses [2]. Our review showed that a variety of methods are used to synthesize evidence from networks of trials, including both IPD-NMAs and MAIC approaches. Indirect comparison methods using IPD have been used in a wide range of clinical disciplines, as have NMAs modeling aggregated data [61, 62]. The majority of the IPD networks applied Bayesian hierarchical models, which is also preferred in NMAs with aggregated data [4, 63]. Similar to IPD meta-analyses [2], one-stage analyses dominated among the statistical approaches. For IPD alone or in combination with aggregated data, models have been developed for dichotomous and continuous outcomes, whereas for the combination of IPD with aggregated data, models also exist for time-to-event data. However, the statistical code is only rarely available to the reader, which was also observed by Sobieraj et al. [61] in NMAs with aggregated data. In agreement with aggregated data NMAs [4, 62], most IPD networks included at least one closed loop. Although the identified IPD-NMAs have been recently published and IPD can be used to assess and adjust for differences in effect modifiers across treatment comparisons avoiding aggregation bias, our findings on consistency agree with findings on aggregated data NMAs [4, 64, 65]. For a review of methods to assess the consistency assumption with an application to an empirical IPD-NMA, we encourage the readers to consult Donegan et al. [66].

Consistent with aggregated NMAs [62], almost half of the 33 empirical IPD indirect comparisons included a network diagram. Among the 33 identified empirical networks, the typical IPD network had a dichotomous, objective primary outcome, compared pharmacological and placebo/control interventions, and involved five interventions and ten trials. Nikolakopoulou et al. [4] indicated that the typical network with aggregated data had a dichotomous, semi-objective primary outcome, compared pharmacological and placebo/control interventions, involved six interventions, and was informed by 21 trials in their scoping review. This difference may be because the conduct of an IPD indirect comparison is resource-intensive and because IPD allows the assessment of more targeted clinical questions, where fewer studies are available. In the retrieved IPD indirect comparisons, no study reported reasons for missing or incomplete IPD, which was also underreported in IPD reviews for meta-analyses [2]. In contrast to NMAs modeling aggregated data, half of the IPD studies were industry-sponsored (27 % vs. 46 %) [61].

One in three empirical approaches used the MAIC method to model IPD. In contrast to IPD-NMAs, both MAIC and STC provide more targeted comparison results, and consider the outcomes observed in the treatments of interest directly. As such, these methods produce a comparison of outcomes based on two specific arms of the available trials reflecting what may have been observed if the treatments had come from the same randomized trial, whereas the remaining treatment comparators involved in the network of trials are analyzed alongside the selected treatments of interest. The advantage of MAIC and STC methods is that they may be used when NMA is impossible, serving as an alternative approach to NMA. However, caution is needed, as these methods are based on the assumption that the studies should have the same clinical characteristics and they do not account for reasons for potential differences across trials examining the treatments of interest.

### Limitations

One limitation of our study is our focus on the presentation and description of methods, characteristics, and reporting of indirect comparison methods with IPD without assessment of the quality of included papers or the methods themselves. However, scoping reviews typically do not include assessment of the risk of bias [13]. Another limitation is our reliance on information reported in the identified articles; as such, we may have missed important methods that were omitted from the authors’ reports, even if these were appropriately applied in their studies. For example, in the 33 empirical networks we included eight methodological articles and one review with empirical examples, where key reporting items may be missing due to space constraints. An additional limitation is that we may not have retrieved all indirect comparison methods with IPD, as some studies may not have been indexed using the search terms we used. However, we believe that our sample is representative of the indirect comparison methods applied in the medical literature, and most of our results are comparable with previous reviews of NMAs using aggregated data, as well as with the results of scoping reviews on IPD meta-analyses.

Previous scoping reviews of NMAs have also shown inadequate reporting [4, 61, 64, 67]. Hence, it is imperative that guidelines are developed to improve the quality of reporting in IPD-NMAs. Further research is also needed to assess the properties and performance of the various indirect comparison methods modeling IPD.

## Conclusions

This is the first scoping review that we are aware of focusing on methods for performing indirect comparisons with IPD, describing also the methodological and reporting characteristics of empirical networks in healthcare. To date, one in three approaches used to model IPD in connected networks of evidence disregarded patient randomization and between-study heterogeneity, considering only information from treatments of interest as if they had come from the same randomized trial. Key methodological and reporting elements (e.g., evaluation of the consistency assumption, existence of a study protocol) were frequently missing, even for networks of trials published in high impact journals. The impact of failing to consider and report important methodological aspects may result in erroneous clinical decisions. It is of paramount importance that reporting of IPD-NMAs is improved and that investigators are aware of the properties of the various indirect methods using IPD before applying them.

## Declarations

### Ethics approval and consent to participate

Not applicable.

### Consent for publication

Not applicable.

### Availability of data and materials

The data supporting our findings are available at Additional file 2. This is the data abstraction file we used to extract information from all eligible studies with individual patient data indirect comparison methods.

## Additional files

Additional file 1:
**Appendix 1.** Literature search for MEDLINE. **Appendix 2.** Data abstraction process from identified studies. **Appendix 3.** Studies excluded during the screening process. **Appendix 4.** Characteristics of the identified indirect comparisons using individual patient data. **Appendix 5.** Epidemiological and descriptive statistics of the identified networks. **Appendix 6.** Reporting characteristics of the identified empirical networks, including unpublished data provided by study authors. **Appendix 7.** Distribution of the number of trials and treatment groups in a network, as well of number of outcomes assessed in indirect comparison methods with individual patient data. **Appendix 8.** Distribution of the number of patients in a network. **Appendix 9.** Included IPD indirect comparison studies. References in Additional file 1. (DOCX 219 kb) Additional file 2: Data abstraction file used to extract information from all eligible studies with individual patient data indirect comparison methods. (XLSX 52 kb)

## Abbreviations

IPD: individual patient data
IQR: interquartile ranges
ISPOR: International Society for Pharmacoeconomics and Outcomes Research
MAIC: matching adjusted indirect comparison
NMA: network meta-analyses
PRESS: peer review of electronic search strategies
PRISMA: preferred reporting items for systematic reviews and meta-analyses
RCT: randomized clinical trial
STC: stimulated treatment comparison
