# Supplementary material for: A scoping review of indirect comparison methods and applications using individual patient data
Source: BMC Med Res Methodol. 2016 Apr 27;16:47. doi: 10.1186/s12874-016-0146-y (PMC4847203; doi:10.1186/s12874-016-0146-y)
Supplement: Additional file 1: — Appendix 1. Literature search for MEDLINE. Appendix 2. Data abstraction process from identified studies. Appendix 3. Studies excluded during the screening process. Appendix 4. Characteristics of the identified indirect comparisons using individual patient data. Appendix 5. Epidemiological and descriptive statistics of the identified networks. Appendix 6. Reporting characteristics of the identified empirical networks, including unpublished data provided by study authors. Appendix 7. Distribution of the number of trials and treatment groups in a network, as well of number of outcomes assessed in indirect comparison methods with individual patient data. Appendix 8. Distribution of the number of patients in a network. Appendix 9. Included IPD indirect comparison studies. References in Additional file 1. (DOCX 219 kb) [file 12874_2016_146_MOESM1_ESM.docx]

Appendix 1-9

[Appendix 1. Literature search for MEDLINE 2](#_Toc445060698)

[Appendix 2. Data abstraction process from identified studies 3](#_Toc445060699)

[General study characteristics 3](#_Toc445060700)

[Characteristics of identified methodologies 3](#_Toc445060701)

[Characteristics of empirical studies 5](#_Toc445060702)

[Appendix 3. Studies excluded during the screening process 7](#_Toc445060703)

[Appendix 4. Characteristics of the identified indirect comparisons using individual patient data 12](#_Toc445060704)

[Appendix 5. Epidemiological and descriptive statistics of the identified networks. 17](#_Toc445060705)

[Appendix 6. Reporting characteristics of the identified empirical networks, including unpublished data provided by study authors. 19](#_Toc445060706)

[Appendix 7. Distribution of the number of trials and treatment groups in a network, as well of number of outcomes assessed in indirect comparison methods with individual patient data. 21](#_Toc445060707)

[Appendix 8. Distribution of the number of patients in a network. 22](#_Toc445060708)

[Appendix 9. Included IPD indirect comparison studies 23](#_Toc445060709)

[References in additional file 1 27](#_Toc445060710)

# Appendix 1. Literature search for MEDLINE

| Database: Ovid MEDLINE(R) In-Process & Other Non-Indexed Citations and Ovid MEDLINE(R) <1946 to Present> Search Strategy:  --------------------------------------------------------------------------------  1 (IPD adj3 (NMA or NMAs or MTC or MTCs or MAIC or MAICs)).tw.  2 (individual patient* adj3 (data or evidence)).tw.  3 (individual participant* adj3 (data or evidence)).tw.  4 IPD.tw.  5 (disaggregat* adj3 data).tw.  6 or/2-5  7 ((network* or network-based) adj3 (meta-analy* or metanaly* or metaanaly* or met analy*)).tw.  8 ((network* or network-based) adj (MA or MAs)).tw.  9 ((MTC or MTCs) adj3 (meta-analy* or metanaly* or metaanaly* or met analy*)).tw.  10 ((mixed treatment* or multiple treatment*) adj3 (compar* or meta-analy* or metanaly* or metaanaly* or met analy*)).tw.  11 ((indirect* or mixed) adj2 compar*).tw.  12 (NMA or NMAs or MTC or MTCs or MAIC or MAICs).tw.  13 or/7-12  14 6 and 13  15 1 or 14  16 (comment or editorial or interview or letter or news).pt.  17 15 not 16  18 exp Animals/ not (exp Animals/ and Humans/)  19 17 not 18  *************************** |
| --- |

# Appendix 2. Data abstraction process from identified studies

## General study characteristics

To describe the general characteristics of all eligible studies, we extracted the name of the first author, the year and journal of publication, and the discipline of the journal according to the Web of Science citation index. We categorized each manuscript by discipline according to the content of the text and the medical area studied in the indirect comparison method (if applicable) to determine disciplines with IPD evidence supporting clinical recommendations. We abstracted country according to the affiliation of the first author, the country of studies included in the indirect comparison method (if applicable), the study title, and the funding source. Each study was categorized according to its funding source as industry-sponsored, publicly sponsored, non-sponsored, or unreported,^1^ to capture whether IPD indirect comparisons require funding to be conducted. We also classified each article using the following categories: methodological, application, methodological/review, or application/protocol article.

## Characteristics of identified methodologies

For each methodological paper, we summarized the proposed methods and models for the IPD indirect comparisons along with their properties to help investigators choosing an IPD method. More specifically, we abstracted the type of data synthesized in the model (i.e., combination of IPD and aggregated data or IPD alone), the statistical framework (i.e., frequentist or Bayesian), the indirect comparison methodology, the type of trial design modeled, the type of outcome data, and the steps required for the IPD indirect comparison (i.e., one-stage or two-stage process).

To describe the indirect comparison methods used and preferred to synthesize IPD, for each application paper we captured the IPD methodology applied, reasons for the choice of analysis, the software used, whether the code for the analysis was provided, and whether important differences were identified if both IPD and aggregated data methods were applied. When different treatment doses were included in the network, we also recorded whether a particular method was applied to account for the relationship between treatment and dose.^2^ The potential dependence of treatment-effects and drug dose is particularly important when comparisons between interventions vary in doses. We also captured the type of model applied (i.e., fixed-effect or random-effects). When a random-effects model was applied, we abstracted the prior (in a Bayesian environment: informative, minimally informative, or non-informative, as stated in the paper) or estimator for between-study variance. There is a wide variety of estimators and priors for the between-study variance, the selection of which may importantly impact on the meta-analysis results.^3 4^ For full networks, we also captured whether the consistency assumption was assessed and extracted the method used for its evaluation.^5^ Methods for ranking treatment effectiveness or safety, such as probability of being the best, and statistical techniques applied for missing participant data were also recorded. Where more than one approach was used to combine data in the network, we abstracted the methods used to compare approaches. We used Preferred reporting items for systematic reviews and meta-analyses (PRISMA) for IPD and NMAs, as well the International Society for Pharmacoeconomics and Outcomes Research (ISPOR) to guide data abstraction, where most of the aforementioned items are considered critical and should be reported in each meta-analysis (see data abstraction form in Additional File 2). ^6-8^ Finally, to assess whether the presentation of results varied we abstracted methods used to report the summary estimates of treatment effect, and we documented whether a network plot was consistently presented across empirical applications. The use of a network plot is particularly important to show the amount and structure of evidence used in an indirect comparison method, especially when data are not provided or comprehensively described.

During the data abstraction process, discrepancies were resolved by discussion or involvement of a third reviewer (SES or ACT). We contacted the corresponding authors via email to obtain additional information about eligible conference abstracts and/or data reported in the included studies with up to 2 reminders. Specifically, we contacted authors to ask for additional details about the process the authors used to identify IPD (e.g., via collaborative research group), the methods used to collect IPD in systematic reviews, and the proportion of contacted authors who provided IPD. If an identified study did not include functioning email addresses for authors, we searched online for alternative contact information. If we failed to identify their email, we searched Google to identify the email of the first, last or the next in order author as presented in the manuscript.

## Characteristics of empirical studies

For each application paper, we abstracted reasons for applying an IPD method, to capture the authors’ views on how IPD may be helpful in indirect comparisons. To assess transparency according to the for IPD guidelines and identify the most frequently used methods to obtain IPD,^6^ we abstracted whether a study protocol existed, time between published protocol and published review, the process used to identify IPD (e.g., collaborative research group, systematic review), the methods used to collect IPD (e.g., mail, email), the number of reminders, the proportion of contacted authors who shared their IPD, the reason for missing any IPD, whether authors requested IPD from all eligible studies or just a subset, time needed to collect, clean, and analyze IPD, and the primary outcome (see Additional File 2). When the primary outcome was not clearly stated, we selected the outcome that met one of the following criteria in the order presented: (1) the outcome listed in the title; (2) the outcome listed in the objectives; (3) the most serious clinical outcome among all studied outcomes; or (4) if the most important outcome was unclear (e.g., the same outcome was reported using both binary and continuous data), the first outcome reported in the text.^9 10^ We categorized each outcome as relating to safety or effectiveness and as objective, semi-objective, or subjective to directly compare the results with the results of previous scoping reviews on aggregated data NMA.^11^ We abstracted additional information on the outcomes , including the total number of outcomes assessed and the number of outcomes that used an IPD methodology to evaluate to which extent each study uses the abstracted IPD. We also extracted the type of outcome data synthesized (e.g., continuous), and the effect measure preferred to analyze IPD (e.g., mean difference). To assess whether access IPD is becoming more challenging requiring legal agreements, we captured if any information was reported on this issue (see abstraction form in Additional File 2). Examples with real life data included in methodological and review papers were considered as applications.

Analogous to previous scoping reviews for NMAs with aggregated data ^11 12^ for describing the network geometry, each network was categorized as a full network (with at least one closed loop) or a tree-shaped network (with no closed loops, including for example star-shaped networks). We abstracted the general characteristics of each network, including the number of trials and patients in the network, NMA comparisons, and IPD trials in the network, as well the number of multi-arm trials. We also recorded the number of competing treatments in the network, the number of patients and type of reference treatment (i.e., active intervention or placebo/control) as reported in each paper. When the reference treatment was not clearly stated in the text, and a placebo was included in the network, we chose placebo as the reference treatment. We categorized the networks according to the included treatment comparisons as pharmacological interventions versus placebo or control, pharmacological versus pharmacological, or non-pharmacological versus any intervention, as defined elsewhere.^11 13^ This information was collected to compare the geometry between networks with IPD and networks with aggregated data from previous reviews,^11 14-16^ and to identify potential differences across the different data types.

# Appendix 3. Studies excluded during the screening process

| **ID** | **Article** | **Reasons for exclusion** |
| --- | --- | --- |
| ***Excluded after title and abstract review*** | | |
| 1 | Achana F, Hubbard S, Sutton A, Kendrick D, Cooper N. An exploration of synthesis methods in public health evaluations of interventions concludes that the use of modern statistical methods would be beneficial. J Clin Epidemiol. 2014;67:376-90 | Not an indirect comparison method with IPD |
| 2 | Alinaghi AY, Jackson PJ, Liu QJ, Wang WW. Joint Mixing Vector and Binaural Model Based Stereo Source Separation. Ieee-Acm Transactions on Audio Speech and Language Processing. 2014;22:1434-48 | Not an indirect comparison method with IPD |
| 3 | Andrews RM, Counahan ML, Hogg GG, McIntyre PB. Effectiveness of a publicly funded pneumococcal vaccination program against invasive pneumococcal disease among the elderly in Victoria, Australia. Vaccine. 2004;23:132-8 | Not an indirect comparison method with IPD |
| 4 | Bath PM, Gray LJ. Systematic reviews as a tool for planning and interpreting trials. Int J Stroke. 2009;4:23-7 | Not an indirect comparison method with IPD |
| 5 | Burdett S, Rydzewska LH, Tierney JF, Pignon JP. Pre-operative chemotherapy improves survival and reduces recurrence in operable non-small cell lung cancer: Preliminary results of a systematic review and metaanalysis of individual patient data from 13 randomised trials. Journal of Thoracic Oncology. 2011;2):S374-S5 | Not an indirect comparison method with IPD |
| 6 | Castellucci LA, Cameron C, Gal GL, Rodger MA, Coyle D, Wells PS, et al. Efficacy and safety outcomes of oral anticoagulants and antiplatelet drugs in the secondary prevention of venous thromboembolism: Systematic review and network meta-analysis. BMJ (Online). 2013;347 | Not an indirect comparison method with IPD |
| 7 | Chen YL, Yu J, Zhang WJ, Zhao Y, Zhang YT, Wang M, et al. An introduction to evidence-based medicine glossary VII. [Chinese]. Chinese Journal of Evidence-Based Medicine. 2009;9:1272-6 | Not an indirect comparison method with IPD |
| 8 | Dias S, Sutton AJ, Welton NJ, Ades AE. Evidence Synthesis for Decision Making 3: Heterogeneity Subgroups, Meta-Regression, Bias, and Bias-Adjustment. Medical Decision Making : an International Journal of the Society for Medical Decision Making. 2013;33:618-40 | Not an indirect comparison method with IPD |
| 9 | Fleeman N, Bagust A, McLeod C, Greenhalgh J, Boland A, Dundar Y, et al. Pemetrexed for the first-line treatment of locally advanced or metastatic non-small cell lung cancer. Health Technol Assess. 2010;14:47-53 | Not an indirect comparison method with IPD |
| 10 | Glenny AM, Altman DG, Song F, Sakarovitch C, Deeks JJ, D'Amico R, et al. Indirect comparisons of competing interventions. Health technology assessment (Winchester, England). 2005;9:1-134, iii-iv | Not an indirect comparison method with IPD |
| 11 | Krause MS, Lutz W. How we really ought to be comparing treatments for clinical purposes. Psychotherapy. 2006;43:359-61 | Not an indirect comparison method with IPD |
| 12 | Kyrgiou M, Salanti G, Pavlidis N, Paraskevaidis E, Ioannidis JPA. Survival benefits with diverse chemotherapy regimens for ovarian cancer: Meta-analysis of multiple treatments. J Natl Cancer Inst. 2006;98:1655-63 | Not an indirect comparison method with IPD |
| 13 | Larkin J, Paine A, Foley G, Mitchell SA, Chen C. First-line treatment in the management of advanced renal cell carcinoma: Systematic review and network meta-analysis. European Journal of Cancer. 2013;49:S656 | Not an indirect comparison method with IPD |
| 14 | Nordmann AJ, Kasenda B, Briel M. Meta-analyses: what they can and cannot do. Swiss Medical Weekly. 2012;142:w13518 | Not an indirect comparison method with IPD |
| 15 | Olkin I, Sampson A. Comparison of meta-analysis versus analysis of variance of individual patient data. Biometrics. 1998;54:317-22 | Not an indirect comparison method with IPD |
| 16 | Ouwens M, Philips Z. How to make use of available survival evidence in an indirect comparison. Value in Health. 2009;12 (7):A388 | Not an indirect comparison method with IPD |
| 17 | Papanicolaou S, Kontodimas S, Syriopoulou V, Tsolia M, Theodoridou M, Strutton DR, et al. Clinical and economic benefits of national immunization with the 13-valent compared to 7- and 10-valent pneumococcal conjugate vaccines in Greece. Value in Health. 2009;12 (7):A423-A4 | Not an indirect comparison method with IPD |
| 18 | Saramago P, Manca A, Sutton AJ. Deriving Input Parameters for Cost-Effectiveness Modeling: Taxonomy of Data Types and Approaches to Their Statistical Synthesis. Value in Health. 2012;15:639-49 | Not an indirect comparison method with IPD |
| 19 | Sculier JP. Role of adjuvant chemotherapy. Radiotherapy and Oncology. 2011;99:S79-S80 | Not an indirect comparison method with IPD |
| 20 | Singh JA, Sloan JA, Atherton PJ, Smith T, Hack TF, Huschka MM, et al. Preferred roles in treatment decision making among patients with cancer: a pooled analysis of studies using the Control Preferences Scale. American Journal of Managed Care. 2010;16:688-96 | Not an indirect comparison method with IPD |
| 21 | Sutton A, Ades AE, Cooper N, Abrams K. Use of indirect and mixed treatment comparisons for technology assessment. PharmacoEconomics. 2008;26:753-67 | Not an indirect comparison method with IPD |
| 22 | Sutton AJ, Cooper NJ, Jones DR. Formalising the use of evidence synthesis to designing future research coherently and efficiently: a framework proposal. Poster presentation at the 16th Cochrane Colloquium: Evidence in the era of globalisation; 2008 Oct 3-7; Freiburg, Germany [abstract]. Zeitschrift fur Evidenz, Fortbildung und Qualitat im Gesundheitswesen. 2008;102:45 | Not an indirect comparison method with IPD |
| 23 | Sutton AJ, Higgins JPI. Recent developments in meta-analysis. Stat Med. 2008;27:625-50 | Not an indirect comparison method with IPD |
| 24 | Trikalinos TA, Segal JB, Boyd CM. Addressing Multimorbidity in Evidence Integration and Synthesis. J Gen Intern Med. 2014;29:661-9. | Not an indirect comparison method with IPD |
| 25 | Woods BS, Hawkins N, Scott DA. Network meta-analysis on the log-hazard scale, combining count and hazard ratio statistics accounting for multi-arm trials: A tutorial. BMC Med Res Methodol. 2010;10 | Not an indirect comparison method with IPD |
| 26 | Zinkstok SM, Vergouwen MDI, Engelter ST, Lyrer PA, Bonati LH, Arnold M, et al. Safety and functional outcome of thrombolysis in dissection-related ischemic stroke: A meta-analysis of individual patient data. Stroke. 2011;42:2515-20 | Not an indirect comparison method with IPD |
| ***Excluded after full-text review*** | | |
| 27 | Ali S, Mealing S, Hawkins N, Lescrauwaet B, Bjork S, Mantovani L, et al. The use of individual patient-level data (IPD) to quantify the impact of pretreatment predictors of response to treatment in chronic hepatitis B patients. BMJ Open. 2013;3 | Not an indirect comparison method with IPD |
| 28 | Citrome L, Stahl S, Meng X, Hochfeld M. Efficacy of iloperidone in the short-term treatment of schizophrenia: Meta-analysis of individual patient data from 4 phase III placebo- and active-comparator controlled trials. Neuropsychopharmacology. 2010;35:S98 | Not an indirect comparison method with IPD |
| 29 | Hoaglin DC, Hawkins N, Jansen JP, Scott DA, Itzler R, Cappelleri JC, et al. Conducting Indirect-Treatment-Comparison and Network-Meta-Analysis Studies: Report of the ISPOR Task Force on Indirect Treatment Comparisons Good Research Practices: Part 2. Value in Health. 2011;14:429-37 | Not an indirect comparison method with IPD |
| 30 | Moore RA, Derry S, Straube S, Ireson-Paine J, Wiffen PJ. Faster, higher, stronger? Evidence for formulation and efficacy for ibuprofen in acute pain. Pain. 2014;155:14-21 | Not an indirect comparison method with IPD |
| 31 | Puhan MA, Bachmann LM, Kleijnen J, Ter Riet G, Kessels AG. Inhaled drugs to reduce exacerbations in patients with chronic obstructive pulmonary disease: a network meta-analysis. BMC Medicine. 2009;7:2 | Not an indirect comparison method with IPD |
| 32 | Reitsma JB, Moons KGM, Bossuyt PMM, Linnet K. Systematic reviews of studies quantifying the accuracy of diagnostic tests and markers. Clinical Chemistry. 2012;58:1534-45 | Not an indirect comparison method with IPD |
| 33 | Ross SD. Trends in meta-analysis. Drug Information Journal. 2009;43:171-6 | Not an indirect comparison method with IPD |
| 34 | Salazar Lindo E, Lehert P, Baumer P. The first mixed treatment comparison in acute diarrhoea in children (individual patient data meta-analysis). Journal of Pediatric Gastroenterology and Nutrition. 2009;48:E30-E1 | Not an indirect comparison method with IPD |
| 35 | Sutton AJ, Cooper NJ, Jones DR. Evidence synthesis as the key to more coherent and efficient research. BMC Medical Research Methodology. 2009;9:29 | Not an indirect comparison method with IPD |
| 36 | Skoetz N, Trelle S, Rancea M, Haverkamp H, Diehl V, Engert A, et al. Effect of initial treatment strategy on survival of patients with advanced-stage Hodgkin's lymphoma: a systematic review and network meta-analysis. Lancet Oncology. 2013;14:943-52 | Not an indirect comparison method with IPD |
| 37 | Wedzicha JA, Dahl R, Buhl R, Schubert-Tennigkeit A, Chen H, D'Andrea P, et al. Pooled safety analysis of the fixed-dose combination of indacaterol and glycopyrronium (QVA149), its monocomponents, and tiotropium versus placebo in COPD patients. Respir Med. 2014;108:1498-507 | Not an indirect comparison method with IPD |
| ***Conference abstracts full text not located*** | | |
| 38 | Boucher R, Abrams KR, Crowther MJ, Lambert PC, Wailoo AJ, Latimer NR. Adjusting for treatment switching in clinical trials when only summary data are available - An evaluation of potential methods. Value in Health. 2013;16 (7):A610-A1 | Full-text not located |
| 39 | Chen H, D'Andrea P, Banerji D. Qva149 does not increase the risk of cardioand cerebro-vascular events, pneumonia and exacerbation events compared with placebo: A network meta-analysis acrossmultiple safety databases. Respiration. 2014;87 (6):559 | Full-text not located |
| 40 | Cutter G, Wolinsky JS, Comi G, Ladkani D, Knappertz V, Vainstein A, et al. Comparable clinical and MRI efficacy of glatiramer acetate 40mg/mL TIW and 20mg/mL QD: Results of a systematic review and meta-analysis. Multiple Sclerosis. 2014;1):90-1 | Full-text not located |
| 41 | Kim H, Gurrin L, Liew D. Evidence requirements in Australia for drug reimbursement: The survival of cost minimisation analysis. Value in Health. 2012;15 (7):A663 | Full-text not located |
| 42 | Saramago P, Weatherly H, Manca A, Sculpher MJ, MacPherson H. Comparative effectiveness of active versus sham acupuncture versus usual care in the management of chronic, non-cancer pain in primary care. Value in Health. 2013;16 (7):A556-A7 | Full-text not located |
| 43 | Wilson JL, Standfield L, Paech D, Sunduram M, Mulani P. Comparative effectiveness of Adalimumab and Etanercept in patients with chronic plaque psoriasis. Australasian Journal of Dermatology. 2012;53:54 | Full-text not located |
| 44 | Woods B, Hawkins N, Paracha N, Sculpher M. Network meta-analysis comparing four drug eluting stents at multiple time points. EuroIntervention. 2010;6 | Full-text not located |

# Appendix 4. Characteristics of the identified indirect comparisons using individual patient data

Abbreviations: MAIC: matching adjusted indirect comparison, STC: simulated treatment comparison, NR: Not reported, NA: Not applicable, BHM: Bayesian hierarchical model, MR: meta-regression model, MC: mixed comparison method

| Study | Method (1-stage/ 2-stage analysis) | Study design | Type of data | Shape of network | #loops | #patients in IPD trials | #patients in the network | Range of trials in the NMA comparisons | Range of patients in the NMA comparisons | Primary Outcome (effectiveness/ safety) | #outcomes in total (#outcomes applied an IPD indirect method) | #patients in the common treatment |
| --- | --- | --- | --- | --- | --- | --- | --- | --- | --- | --- | --- | --- |
| *Methodological/Simulation studies* | | | | | | | | | | | | |
| Jansen 2012 [1] | BHM  (1-stage) | RCTs | IPD only & IPD+AD | Tree-shaped Network | 0 | {1800, 1800} | {6000, 6000} | {(8, 8), (15, 15)} | {(3000, 3000), (3000, 3000)} | NA (NA) | NA (NA) | {3000, 3000} |
| Signorovitch 2013 (a) [33] | MAIC | RCTs | IPD+AD | Tree-shaped Network | 0 | NR | NR | (1, 1) | NR | NA | NA (NA) | NA |
| *Methodological articles* | | | | | | | | | | | | |
| Caro 2010 [37] | STC | NA | IPD+AD | NA | NA | NA | NA | NA | NA | NA | NA (NA) | NA |
| Donegan 2012 [4, 5, 46] | BHM  (1-stage) | RCTs | IPD only | Full network^3^ | 4 | 3874 | 3874 | (2, 13) | (782, 2225) | Treatment success of uncomplicated Plasmodia falciparum malaria in children at day 28 (Effectiveness) | 1 (1) | 1381 |
| Donegan 2013 [5, 6] | BHM  (1-stage) | RCTs | IPD+AD | Full network^3^ | 7 | 3344 | 14634 | (1, 13) | (260, 4680) | Treatment success of uncomplicated Plasmodium falciparum malaria at day 28 (Effectiveness) | 1 (1) | 1825 |
| Hong 2014 [7] | BHM  (1-stage) | RCTs | IPD only | Full network^3^ | 1 | 5417 | 5417 | (1, 7) | (269, 2364) | Glycosylated hemoglobin -HbA1c (Effectiveness) | 2 (2) | 2802 |
| Nixon 2014 [34] | Extended MAIC | RCTs | IPD+AD | Full network | 2 | 1556 | 4945 | (1, 2) | (721, 1556) | Proportion of patients free from relapses (Effectiveness) | 1 (1) | 1907 |
| Saramago 2014 [8] | BHM  (1-stage) | RCTs | IPD+AD | Full network^3^ | 1 | 841 | 1946 | (1, 7) | (121, 1072) | Time to healing (Effectiveness) | 1 (1) | 850 |
| Saramago 2012 [2, 3] | BHM  (1-stage) | RCTs, Non-RCTs, CBA | IPD only & IPD+AD | Full network^3^ | 4 | 3886 | 11479 | (1, 4) | (79, 3128) | Provision of functioning smoke alarm safety equipment to prevent burns in children (Effectiveness) | 1 (1) | 3340 |
| Thom 2014 [9] | BHM  (1-stage) | RCTs + observational | IPD+AD | Full network^3^ | 2 | 146 | 1110 | (1, 4) | (10, 387) | Change in 6-minute walk distance (Effectiveness) | 7 (7) | 493 |
| Signorovitch 2010 [35, 36] | MAIC | RCTs | IPD+AD | Tree-shaped Network | 0 | 591 | 921 | (1, 2) | (330, 591) | Proportion of patients who achieved a >=75% improvement in PASI score at 12 weeks (Effectiveness) | 3 (3) | 369 |
| *Methodological/Review articles* | | | | | | | | | | | | |
| Ishak 2015 [38] | MAIC & STC | NA | IPD+AD | NA | NA | NA | NA | NA | NA | NA | NA (NA) | NA |
| Veroniki 2014 [10] | BHM  (1-stage) | RCTs | IPD only & IPD+AD | Full network^3^ | 1 | 3822 | 3822 | (1, 4) | (52, 3273) | HIV risk reduction (Effectiveness) | 1 (1) | 234 |
| *Application/Protocol articles^2^* | | | | | | | | | | | | |
| Ruifrok 2014 [11, 12] | Bucher  (2-stage) | RCTs | IPD only | Tree-shaped Network | 0 | NR | NR | NR | NR | Maternal fatal complications (Safety) | 3 (NR) | NR |
| *Application articles* | | | | | | | | | | | | |
| Bergvall 2013 [43] | MAIC | RCTs | IPD+AD | Tree-shaped Network | 0 | NR | NR | (1, 2) | NR | Time to progression to severe disability health states in pts. with relapsing-remitting MS (Effectiveness) | 1 (1) | NR |
| Blanchard 2011 [19, 20] | BHM  (1-stage) | RCTs | IPD only | Full network^3^ | 4 | 23000 | 23000 | (1, 45) | (127, 8119) | Survival of neck and head cancer (Effectiveness) | 1 (1) | NR |
| CNT Collaboration 2013 [26] | MC  (2-stage) | RCTs | IPD+AD | Full network | 3 | 214164 | 258951 | (20, 113) | (21398, 73635) | Major vascular events (Safety) | 3 (3) | 175 |
| Cope 2012 (a) [21] | BHM  (1-stage) | RCTs | IPD only | Full network^3^ | 5 | 3292 | 3292 | (1, 2) | (564, 1370) | Forced expiratory volume in 1 second at 6 months (Effectiveness) | 3 (3) | 918 |
| Cope 2012 (b) [22] | BHM  (1-stage) | RCTs | IPD only | Full network^3^ | 6 | 4191 | 4191 | (1, 3) | (641, 1929) | Forced expiratory volume in 1 second at week 12 (Effectiveness) | 2 (2) | 1716 |
| Daniels 2012 [23] | MR  (2-stage) | RCTs | IPD+AD | Full network^3^ | 3 | 1717 | 3287 | (1, 8) | (111, 820) | Heavy menstrual bleeding (Effectiveness) | 3 (3) | 899 |
| Ellis 2014 (a) [27] | BHM  (1-stage) | RCTs | IPD+AD | Tree-shaped Network | 0 | 5643 | 17876 | (1, 3) | (1952, 6961) | Incidence of vertebral fractures (Effectiveness) | 1 (1) | 7406 |
| Ellis 2014 (b) [28] | BHM  (1-stage) | RCTs | IPD+AD | Tree-shaped Network | 0 | 5643 | 29267 | (1, 5) | (1952, 12147) | Incidence of non-vertebral fractures (Effectiveness) | 1 (1) | 12064 |
| Goodacre 2014 [29] | BHM  (1-stage) | RCTs + quasi-RCTs | IPD+AD | Tree-shaped Network | 0 | 650 | 800 | (4, 6) | (190, 610) | Mortality (Effectiveness) | 2 (2) | 393 |
| Mealing 2014 [30] | BHM  (NA^1^) | RCTs | IPD+AD | Full network^3^ | 2 | 1353 | 4253 | (1, 3) | (65, 1252) | Undetectable viral load after 1 year (Effectiveness) | 1 (1) | 310 |
| Mills 2014 [31] | BHM  (unclear) | RCTs | IPD+AD | Full network^3^ | 8 | NR | 5310 | (1, 5) | (104, 2473) | Adherence to antiretroviral therapy, as defined by trial adherence criteria (Effectiveness) | 2 (2) | 1148 |
| Middleton 2010 [18] | MR  (2-stage) | RCTs | IPD+AD | Full network | 1 | 1864 | 2886 | (1, 11) | (69, 1785) | Dissatisfaction at 12 months (Effectiveness) | 1 (1) | 1342 |
| Palmerini 2014 [32] | BHM  (2-stage) | RCTs | IPD only | Tree-shaped Network | 0 | 8180 | 8180 | (2, 2) | (2944, 5236) | 1-year rate of major adverse cardiac events (Effectiveness) | 2 (1) | 4085 |
| Pignon 2009 [14-17] | Bucher  (2-stage) | RCTs | IPD only | Full network | 1 | 15787 | 15787 | (6, 50) | (861, 9615) | Overall survival of neck and head cancer (Effectiveness) | 6 (6) | 2567 |
| Signorovitch 2011 (a) [36, 40] | MAIC | RCTs | IPD+AD | Tree-shaped Network | 0 | 553 | 1072 | (1, 1) | (519, 553) | Overall survival by 12-months (Effectiveness) | 3 (3) | 540 |
| Signorovitch 2011 (b) [36, 41] | MAIC | RCTs | IPD+AD | Full network | 1 | 528 | 1213 | (1, 2) | (142, 380) | HbA1c change from baseline to week 11 (Effectiveness) | 1 (1) | 221 |
| Signorovitch 2012 [36, 39] | MAIC | RCTs | IPD+AD | Tree-shaped Network | 0 | 143 | 241 | (1, 2) | (98, 214) | Change in CPRS-RS:OS score from baseline to endpoint (Effectiveness) | 1 (1) | 101 |
| Signorovitch 2013 (b) | MAIC | RCTs | IPD+AD | Tree-shaped Network | 0 | 394 | 565 | (1, 1) | (171, 394) | Overall survival (Effectiveness) | 3 (3) | 288 |
| Szegedi 2012 [24] | MR  (2-stage) | RCTs | IPD+AD | Full network^3^ | 17 | 577 | 14861 | (1, 15) | (114, 2404) | Change from baseline PANSS total score (Effectiveness) | 1 (1) | NR |
| Sikirica 2013 [42] | MAIC | RCTs | IPD+AD | Tree-shaped Network | 0 | 218 | 385 | (1, 2) | (167, 218) | Change in ADHD-RS-IV total score (Effectiveness) | 3 (3) | 219 |
| Tudur Smith 2007 [13] | MR  (1-stage) | RCTs | IPD only | Full network | 18 | 6831 | 6831 | (1, 6) | (178, 2116) | Time to treatment failure for partial onset seizures (Effectiveness) | 3 (3) | 1731 |
| Xie 2012 [44, 45] | MAIC | RCTs | IPD+AD | Tree-shaped Network | 0 | 726 | 1415 | (1, 1) | (689, 726) | Virologic response at week 48 (Effectiveness) | 1 (1) | 784 |
| Youdom 2012 [25] | BHM  (1-stage) | RCTs | IPD only | Full network^3^ | 1 | 621 | 621 | (1, 1) | (115, 164) | Response for ACPR recorded on days 14 (Effectiveness) | 1 (1) | 203 |

^1^ The results of a previous IPD meta-analysis were used to inform the prior distribution on a regression coefficient

^2^ Protocols included in this section are not companion reports

^3^ A network plot was available in the paper. In case a network plot was not presented in the manuscript, we designed the network geometry according to the data presented in the tables and appendices.

# Appendix 5. Epidemiological and descriptive statistics of the identified networks.

Table 1. Figures are no. (%) of studies.

| **Characteristic** | **IPD-NMA studies*** | | **MAIC/STC studies*** | | **Total*** |
| --- | --- | --- | --- | --- | --- |
| **Country of first author** | |  | |  | |
| United States | 7 (47) | | 8 (53) | | 15 (41) |
| United Kingdom | 12 (100) | | 0 (0) | | 12 (32) |
| Canada | 1 (33) | | 2 (67) | | 3 (8) |
| France | 3 (100) | | 0 (0) | | 3 (8) |
| Switzerland | 0 (0) | | 2 (100) | | 2 (5) |
| Greece | 1 (100) | | 0 (0) | | 1 (3) |
| Italy | 1 (100) | | 0 (0) | | 1 (3) |
| **Total** | **25 (68)** | | **12 (32)** | | **37 (100)** |
| **Journal of publication**† |  | |  | |  |
| Statistics in Medicine | 4 (100) | | 0 (0) | | 4 (12) |
| Pharmacoeconomics | 0 (0) | | 3 (100) | | 3 (9) |
| (BMC) Systematic Reviews | 2 (100) | | 0 (0) | | 2 (6) |
| BMC Medical Research Methodology | 2 (100) | | 0 (0) | | 2 (6) |
| BMJ | 2 (100) | | 0 (0) | | 2 (6) |
| Current Medical Research and Opinion | 1 (50) | | 1 (50) | | 2 (6) |
| The Lancet | 2 (100) | | 0 (0) | | 2 (6) |
| Value in Health | 2 (100) | | 0 (0) | | 2 (6) |
| (BMC) Malaria Journal | 1 (100) | | 0 (0) | | 1 (3) |
| (BMC) Trials | 1 (100) | | 0 (0) | | 1 (3) |
| Academic Emergency Medicine | 1 (100) | | 0 (0) | | 1 (3) |
| Advances in Therapy | 0 (0) | | 1 (100) | | 1 (3) |
| BMC Pulmonary Medicine | 1 (100) | | 0 (0) | | 1 (3) |
| Journal of Central Nervous System Disease | 0 (0) | | 1 (100) | | 1 (3) |
| Clinical Drug Investigation | 0 (0) | | 1 (100) | | 1 (3) |
| Experimental Hematology & Oncology | 0 (0) | | 1 (100) | | 1 (3) |
| Journal of Clinical Epidemiology | 1 (100) | | 0 (0) | | 1 (3) |
| Journal of Clinical Psychiatry | 1 (100) | | 0 (0) | | 1 (3) |
| Journal of the American College of Cardiology | 1 (100) | | 0 (0) | | 1 (3) |
| Pharmacoepidemiology and Drug Safety | 0 (0) | | 1 (100) | | 1 (3) |
| Radiotherapy and Oncology | 1 (100) | | 0 (0) | | 1 (3) |
| Research Synthesis Methods | 1 (100) | | 0 (0) | | 1 (3) |
| **Total** | **24 (72)** | | **9 (67)** | | **33 (100)** |
| **Discipline of journal**† |  | |  | |  |
| General and internal medicine | 5 (83) | | 1 (17) | | 6 (18) |
| Economics | 2 (40) | | 3 (60) | | 5 (15) |
| Health care sciences and services | 5 (100) | | 0 (0) | | 5 (15) |
| Mathematical and computational biology | 5 (100) | | 0 (0) | | 5 (15) |
| Pharmacology and pharmacy | 0 (0) | | 2 (100) | | 2 (6) |
| Research and experimental medicine | 1 (50) | | 1 (50) | | 2 (6) |
| Cardiac and cardiovascular systems | 1 (100) | | 0 (0) | | 1 (3) |
| Clinical neurology | 0 (0) | | 1 (100) | | 1 (3) |
| Clinical psychology | 1 (100) | | 0 (0) | | 1 (3) |
| Emergency medicine | 1 (100) | | 0 (0) | | 1 (3) |
| Hematology | 0 (0) | | 1 (100) | | 1 (3) |
| Infectious diseases | 1 (100) | | 0 (0) | | 1 (3) |
| Oncology | 1 (100) | | 0 (0) | | 1 (3) |
| Respiratory medicine | 1 (100) | | 0 (0) | | 1 (3) |
| **Total** | **24 (72)** | | **9 (67)** | | **33 (100)** |
| **Funding source** |  | |  | |  |
| Industry-sponsored | 8 (47) | | 9 (53) | | 17 (46) |
| Publicly sponsored | 9 (100) | | 0 (0) | | 9 (24) |
| Funding not reported | 2 (50) | | 2 (50) | | 4 (11) |
| Non-sponsored | 3 (75) | | 1 (25) | | 4 (11) |
| Mixed funding | 3 (100) | | 0 (0) | | 3 (8) |
| **Total** | **25 (68)** | | **12 (32)** | | **37 (100)** |
| **Type of network articles** |  | |  | |  |
| Application | 16 (70) | | 7 (30) | | 23 (62) |
| Methodological | 7 (64) | | 4 (36) | | 11 (30) |
| Methodological/review | 1 (50) | | 1 (50) | | 2 (5) |
| Protocol/application | 1 (100) | | 0 (0) | | 1 (3) |
| **Total** | **25 (68)** | | **12 (32)** | | **37 (100)** |
| **Geographic region of trials included in network**‡ | | | | | |
| Multi-continental | 5 (83) | | 1 (17) | | 6 (18) |
| Africa | 4 (100) | | 0 (0) | | 4 (12) |
| North America | 1 (33) | | 2 (67) | | 3 (9) |
| Not reported | 14 (70) | | 6 (30) | | 20 (61) |
| **Total** | **24 (73)** | | **9 (27)** | | **33 (100)** |
| IPD-NMA=individual patient-data network meta-analysis; MAIC=matching adjusted indirect comparison; STC=simulation treatment comparison.  *Percentages were calculated across the row for IPD-NMA and MAIC/STC, but down the column for the “Total” column. Total number of included studies n = 37. Total number of empirical networks n = 33. Please note that the empirical networks include 8 methodological and 1 review papers.  †The journal discipline was not applicable for 3 conference abstracts,^17-19^ and 1 book chapter.^20^  ‡Two simulation studies^17 21^, one methodological/review article,^22^ and one methodological article^23^ were not considered in this section, as they did not include an application example. | | | | | |

# Appendix 6. Reporting characteristics of the identified empirical networks, including unpublished data provided by study authors.

**Table 2.** Figures are no. (%) of studies.

| **Characteristic** | **IPD-NMA studies*** | **MAIC studies*** | **Total*** |
| --- | --- | --- | --- |
| **Existence of study protocol** |  |  | |
| No/unclear^$^ | 15 (63) | 9 (37) | 24 (73) |
| Yes | 9 (100) | 0 (0) | 9 (27) |
| **Total** | **24 (73)** | **9 (27)** | **33 (100)** |
| **Process to identify IPD eligible studies** | | | |
| Collaborative research group** | 13 (59) | 9 (41) | 22 (67) |
| Systematic review and contacting authors | 9 (100) | 0 (0) | 9 (27) |
| Results from previous IPD-MA | 1 (100) | 0 (0) | 1 (3) |
| Not reported^#^ | 1 (100) | 0 (0) | 1 (3) |
| **Total** | **24 (73)** | **9 (27)** | **33 (100)** |
| **Methods used to collect IPD in systematic reviews†** | | | |
| Email, mail, and telephone | 2 (100) | 0 (0) | 2 (22) |
| Email | 1 (100) | 0 (0) | 1 (11) |
| Email and mail | 1 (100) | 0 (0) | 1 (11) |
| Email, Skype/telephone, face-to-face meetings | 2 (100) | 0 (0) | 2 (22) |
| Not reported^#^ | 3 (100) | 0 (0) | 3 (33) |
| **Total** | **9 (100)** | **0 (0)** | **9 (100)** |
| **Safety/effectiveness** |  |  |  |
| Effectiveness | 22 (71) | 9 (29) | 31 (94) |
| Safety | 2 (100) | 0 (0) | 2 (6) |
| **Total** | **24 (73)** | **9 (27)** | **33 (100)** |
| **Outcome category** |  |  |  |
| Objective | 20 (77) | 6 (23) | 26 (79) |
| Semi-objective | 3 (50) | 3 (50) | 6 (18) |
| Subjective | 1 (100) | 0 (0) | 1 (3) |
| **Total** | **24 (73)** | **9 (27)** | **33 (100)** |
| **Outcome data type** |  |  |  |
| Dichotomous | 14 (82) | 3 (18) | 17 (52) |
| Continuous | 5 (56) | 4 (44) | 9 (27) |
| Rate | 1 (100) | 0 (0) | 1 (3) |
| Time-to-event | 4 (67) | 2 (33) | 6 (18) |
| **Total** | **24 (73)** | **9 (27)** | **33 (100)** |
| **Effect measure** |  |  |  |
| Odds ratio | 11 (100) | 0 (0) | 11 (33) |
| Mean difference | 5 (63) | 3 (37) | 8 (24) |
| Hazard ratio | 4 (67) | 2 (33) | 6 (18) |
| Risk ratio | 1 (33) | 2 (67) | 3 (9) |
| Both odds ratio and risk ratio | 2 (100) | 0 (0) | 2 (6) |
| Standardized mean difference | 0 (0) | 1 (100) | 1 (3) |
| Risk difference | 0 (0) | 1 (100) | 1 (3) |
| Rate ratio | 1 (100) | 0 (0) | 1 (3) |
| **Total** | **24 (73)** | **9 (27)** | **33 (100)** |
| **Type of reference treatment** |  |  |  |
| Placebo/control | 11 (61) | 7 (39) | 18 (55) |
| Active intervention | 13 (87) | 2 (13) | 15 (45) |
| **Total** | **24 (73)** | **9 (27)** | **33 (100)** |
| **Intervention comparison type** |  |  |  |
| Pharmacological vs. placebo/control | 10 (59) | 7 (41) | 17 (52) |
| Non-pharmacological vs. any | 8 (100) | 0 (0) | 8 (24) |
| Pharmacological vs. pharmacological | 6 (75) | 2 (25) | 8 (24) |
| **Total** | **24 (73)** | **9 (27)** | **33 (100)** |
| **Shape of network** |  |  |  |
| Full network | 19 (90) | 2 (10) | 21 (64) |
| Open network | 5 (42) | 7 (58) | 12 (36) |
| **Total** | **24 (73)** | **9 (27)** | **33 (100)** |
| **Network plot presented in the paper** | | | |
| No | 8 (47) | 9 (53) | 17 (52) |
| Yes | 15 (100) | 0 (0) | 15 (45) |
| Not applicable (study protocol) | 1 (100) | 0 (0) | 1 (3) |
| **Total** | **24 (73)** | **9 (27)** | **33 (100)** |
| **Approaches to reporting of summary estimates** | | | |
| Tables, forest plot | 7 (78) | 2 (22) | 9 (27) |
| Tables, other plots‡ | 4 (57) | 3 (43) | 7 (21) |
| Tables, bar plots | 3 (50) | 3 (50) | 6 (18) |
| Tables | 4 (100) | 0 (0) | 4 (12) |
| Tables, forest plot, other plots‡ | 2 (100) | 0 (0) | 2 (6) |
| Bar plots | 0 (0) | 1 (100) | 1 (3) |
| Forest plots | 1 (100) | 0 (0) | 1 (3) |
| Forest plots, other plots‡ | 1 (100) | 0 (0) | 1 (3) |
| Tables, forest plot, bar plots | 1 (100) | 0 (0) | 1 (3) |
| Not applicable (study protocol) | 1 (100) | 0 (0) | 1 (3) |
| **Total** | **24 (73)** | **9 (27)** | **33 (100)** |
| IPD-NMA=individual patient data network meta-analysis; IPD-MA=individual patient data meta-analysis; MAIC=matching adjusted indirect comparison.  *Percentages were calculated across the row for IPD-NMA and MAIC/STC, but down the column for the “Total” column. Total number of included studies n = 37. Total number of empirical networks n = 33. Please note that the empirical networks include 8 methodological and 1 review papers.  **One IPD-NMA had access to IPD from a previous randomized controlled trial conducted by the same authors^24^.  †In total, nine systematic reviews were applied to identify the eligible IPD studies.  ‡Other plot types are probability plots, Kaplan Meier plots, line charts, scatter plots.  ^#^We contacted the study authors when this information was not reported or unclear  ^$^Four methodological papers cited an original empirical study, and these 4 studies provided a reference for their study protocol. | | | |

# Appendix 7. Distribution of the number of trials and treatment groups in a network, as well of number of outcomes assessed in indirect comparison methods with individual patient data.


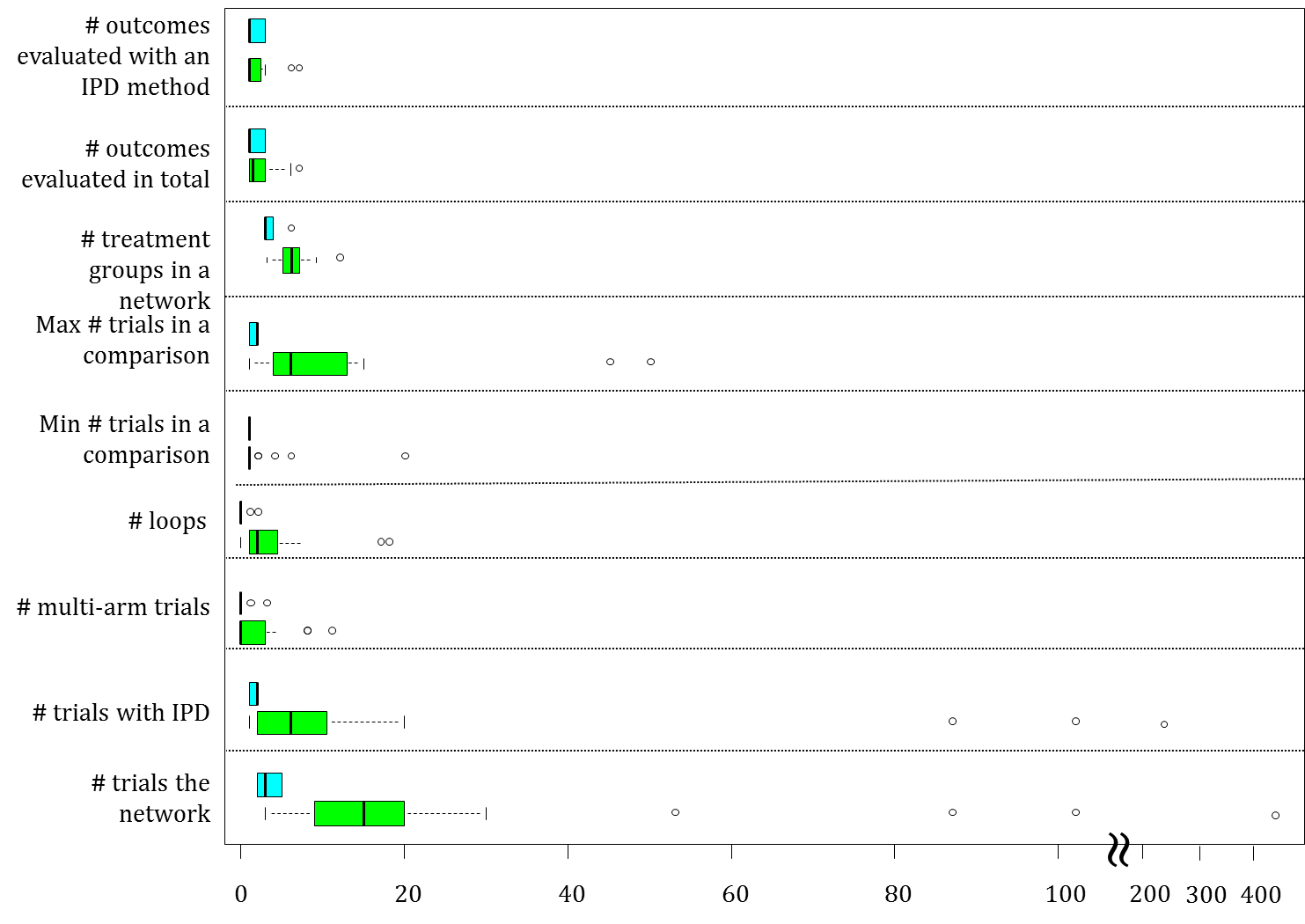


Legend: Green boxplots correspond to IPD-NMAs, and blue boxplots to MAIC methods.

# **Appendix 8. Distribution of the number of patients in a network.**


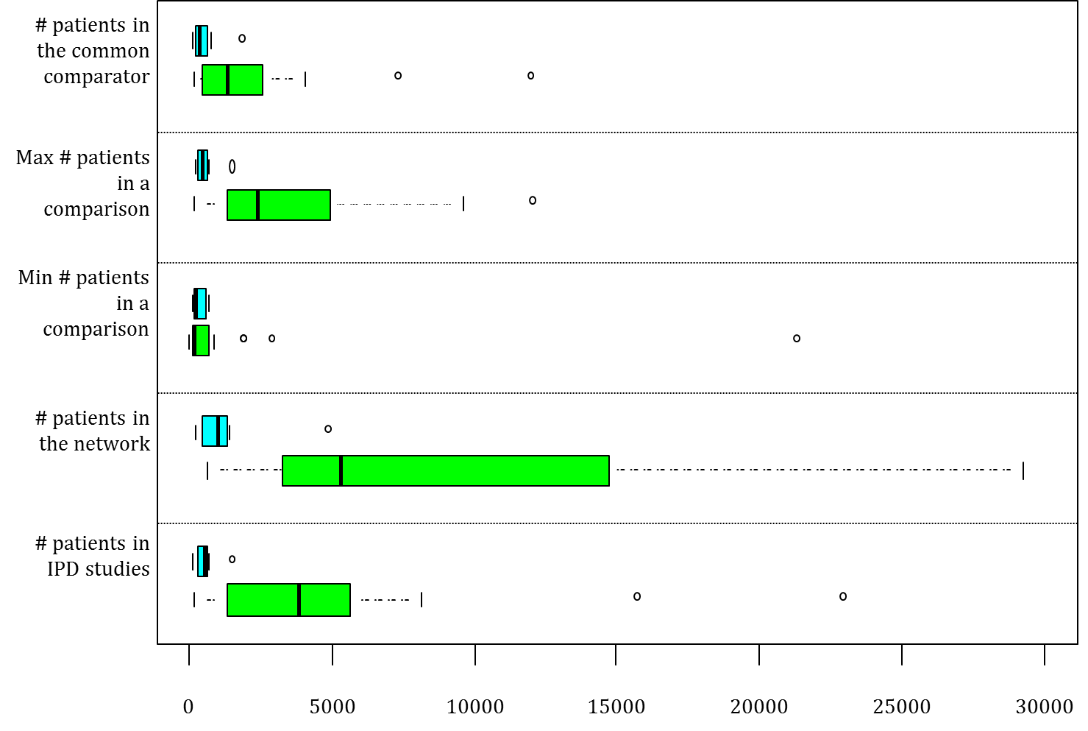


Legend: Green boxplots correspond to IPD-NMA methods, and blue boxplots to MAIC methods.

# **Appendix 9.** Included IPD indirect comparison studies

1. Jansen JP, Cope S: Network meta-analysis of individual and aggregate level data. Value in Health 2012, 15 (4):A159.

2. Saramago P, Sutton AJ, Cooper NJ, Manca A: Mixed treatment comparisons using aggregate and individual participant level data. Statistics in Medicine 2012, 31(28):3516-3536.

3. Saramago P: Methodological issues in the analysis of individual- and aggregate-participant level data for cost effectiveness analysis. University of York; 2012.

4. Donegan S, Williamson P, D'Alessandro U, Smith CT: Assessing the consistency assumption by exploring treatment by covariate interactions in mixed treatment comparison meta-analysis: Individual patient-level covariates versus aggregate trial-level covariates. Statistics in Medicine 2012, 31(29):3840-3857.

5. Donegan S: The value of individual patient data for mixed treatment comparison meta-analysis. University of Liverpool; 2011.

6. Donegan S, Williamson P, D'Alessandro U, Garner P, Smith CT: Combining individual patient data and aggregate data in mixed treatment comparison meta-analysis: Individual patient data may be beneficial if only for a subset of trials. Statistics in Medicine 2013, 32(6):914-930.

7. Hong H, Fu H, Price KL, Carlin BP: Incorporation of individual-patient data in network meta-analysis for multiple continuous endpoints, with application to diabetes treatment. Stat Med 2015.

8. Saramago P, Chuang LH, Soares MO: Network meta-analysis of (individual patient) time to event data alongside (aggregate) count data. BMC Medical Research Methodology 2014, 14:105.

9. Thom HH, Capkun G, Cerulli A, Nixon RM, Howard LS: Network meta-analysis combining individual patient and aggregate data from a mixture of study designs with an application to pulmonary arterial hypertension. BMC Med Res Methodol 2015, 15:34.

10. Veroniki AA, Huedo-Medina TB, Fountoulakis KN: Moving from study-level to patient-level data: individual patient network meta-analysis. Network Meta-Analysis: Evidence Synthesis with Mixed Treatment Comparison: NY: Nova Science Publishers; 2014.

11. Ruifrok AE, Rogozinska E, van Poppel MN, Rayanagoudar G, Kerry S, de Groot CJ, Yeo S, Molyneaux E, McAuliffe FM, Poston L et al: Study protocol: differential effects of diet and physical activity based interventions in pregnancy on maternal and fetal outcomes--individual patient data (IPD) meta-analysis and health economic evaluation. Syst Rev 2014, 3:131.

12. Thangaratinam S, Khan K, Riley R, Kerry S, Jit M, Mol B, Ruifrok A, Coomarasamy A, Rogozinska E, Dodds J: Effects of weight management interventions on maternal and fetal outcomes in pregnancy: Individual patient data (IPD) meta analysis of randomised trials and model based economic evaluation. In. PROSPERO International prospective register of systematic reviews: University of York; 2013.

13. Tudur Smith C, Marson AG, Chadwick DW, Williamson PR: Multiple treatment comparisons in epilepsy monotherapy trials. Trials [Electronic Resource] 2007, 8:34.

14. Pignon JP, Maitre Al, Maillard E, Bourhis J: Meta-analysis of chemotherapy in head and neck cancer (MACH-NC): An update on 93 randomised trials and 17,346 patients. Radiotherapy and Oncology 2009, 92(1):4-14.

15. Pignon JP: Individual patient data meta-analysis and radiotherapy in head and neck and lung cancer. Radiotherapy and Oncology 2010, 96:S54-S55.

16. Pignon JP, Bourhis J, Domenge C, Designe L: Chemotherapy added to locoregional treatment for head and neck squamous-cell carcinoma: three meta-analyses of updated individual data. MACH-NC Collaborative Group. Meta-Analysis of Chemotherapy on Head and Neck Cancer. Lancet 2000, 355(9208):949-955.

17. Pignon JP, le Maitre A, Bourhis J, Group M-NC: Meta-Analyses of Chemotherapy in Head and Neck Cancer (MACH-NC): an update. Int J Radiat Oncol Biol Phys 2007, 69(2 Suppl):S112-114.

18. Middleton LJ, Champaneria R, Daniels JP, Bhattacharya S, Cooper KG, Hilken NH, O'Donovan P, Gannon M, Gray R, Khan KS: Hysterectomy, endometrial destruction, and levonorgestrel releasing intrauterine system (Mirena) for heavy menstrual bleeding: Systematic review and meta-analysis of data from individual patients. BMJ (Online) 2010, 341(7769):379.

19. Blanchard P, Hill C, Guihenneuc-Jouyaux C, Baey C, Bourhis J, Pignon JP: Mixed treatment comparison meta-analysis of altered fractionated radiotherapy and chemotherapy in head and neck cancer. Journal of Clinical Epidemiology 2011, 64(9):985-992.

20. Pignon J: Individual patient data meta-analysis in non-metastatic head & neck and lung cancers: the Gustave Roussy experience. In. Meta-analysis team, Gustave-Roussy, Villejuif, France; 2015.

21. Cope S, Capkun-Niggli G, Gale R, Lassen C, Owen R, Ouwens MJNM, Bergman G, Jansen JP: Efficacy of once-daily indacaterol relative to alternative bronchodilators in COPD: A patient-level mixed treatment comparison. Value in Health 2012, 15(3):524-533.

22. Cope S, Zhang J, Raparla S, Williams J, Jansen J: Comparative efficacy of once-daily indacaterol 75 mug in COPD in terms of forced expiratory volume: A patient level network meta-analysis. Chest 2011, 140 (4 MEETING ABSTRACT).

23. Daniels JP, Middleton LJ, Champaneria R, Khan KS, Cooper K, Mol BW, Bhattacharya S, International Heavy Menstrual Bleeding IPDM-aCG: Second generation endometrial ablation techniques for heavy menstrual bleeding: network meta-analysis. BMJ 2012, 344:e2564.

24. Szegedi A, Verweij P, Van Duijnhoven W, Mackle M, Cazorla P, Karson C, Fennema H: Efficacy of asenapine for schizophrenia: Comparison with placebo and comparative efficacy of all atypical antipsychotics using all available head-to-head randomized trials using meta-analytical techniques. Neuropsychopharmacology 2012, 35:S105.

25. Whegang Youdom S, Samson A, Basco LK, Thalabard JC: Multiple treatment comparisons in a series of anti-malarial trials with an ordinal primary outcome and repeated treatment evaluations. Malaria Journal 2012, 11:147.

26. Coxib, traditional NTC, Bhala N, Emberson J, Merhi A, Abramson S, Arber N, Baron JA, Bombardier C, Cannon C et al: Vascular and upper gastrointestinal effects of non-steroidal anti-inflammatory drugs: meta-analyses of individual participant data from randomised trials. Lancet 2013, 382(9894):769-779.

27. Ellis AG, Reginster JY, Luo X, A GB, Williams R, Sutradhar S, Mirkin S, Jansen JP: Indirect comparison of bazedoxifene vs oral bisphosphonates for the prevention of vertebral fractures in postmenopausal osteoporotic women. Current Medical Research & Opinion 2014, 30(8):1617-1626.

28. Ellis AG, Reginster JY, Luo X, Cappelleri JC, Chines A, Sutradhar S, Jansen JP: Bazedoxifene versus oral bisphosphonates for the prevention of nonvertebral fractures in postmenopausal women with osteoporosis at higher risk of fracture: a network meta-analysis. Value in Health 2014, 17(4):424-432.

29. Goodacre S: Pre-hospital non-invasive ventilation for acute respiratory failure: a systematic review and network meta analysis. Emergency Medicine Journal 2014, 31(9):778-778.

30. Mealing S, Ghement I, Hawkins N, Scott DA, Lescrauwaet B, Watt M, Thursz M, Lampertico P, Mantovani L, Morais E et al: The importance of baseline viral load when assessing relative efficacy in treatment-naive HBeAg-positive chronic hepatitis B: a systematic review and network meta-analysis. Systems Review 2014, 3:21.

31. Mills EJ, Lester R, Thorlund K, Lorenzi M, Muldoon K, Kanters S, Linnemayr S, Gross R, Calderon Y, Amico KR et al: Interventions to promote adherence to antiretroviral therapy in Africa: a network meta-analysis. The Lancet HIV, 1(3):e104-e111.

32. Palmerini T, Sangiorgi D, Valgimigli M, Biondi-Zoccai G, Feres F, Abizaid A, Costa RA, Hong MK, Kim BK, Jang Y et al: Short- versus long-term dual antiplatelet therapy after drug-eluting stent implantation: an individual patient data pairwise and network meta-analysis. J Am Coll Cardiol 2015, 65(11):1092-1102.

33. Signorovitch J, Ayyagari R, Cheng D, Wu EQ: Matching-adjusted indirect comparisons: A simulation study of statistical performance. Value in Health 2013, 16 (3):A48.

34. Nixon R, Bergvall N, Tomic D, Sfikas N, Cutter G, Giovannoni G: No evidence of disease activity: indirect comparisons of oral therapies for the treatment of relapsing-remitting multiple sclerosis. Adv Ther 2014, 31(11):1134-1154.

35. Signorovitch JE, Wu EQ, Yu AP, Gerrits CM, Kantor E, Bao Y, Gupta SR, Mulani PM: Comparative effectiveness without head-to-head trials: a method for matching-adjusted indirect comparisons applied to psoriasis treatment with adalimumab or etanercept. Pharmacoeconomics 2010, 28(10):935-945.

36. Signorovitch JE, Sikirica V, Erder MH, Xie J, Lu M, Hodgkins PS, Betts KA, Wu EQ: Matching-adjusted indirect comparisons: a new tool for timely comparative effectiveness research. Value in Health 2012, 15(6):940-947.

37. Caro JJ, Ishak KJ: No head-to-head trial? simulate the missing arms. Pharmacoeconomics 2010, 28(10):957-967.

38. Ishak KJ, Proskorovsky I, Benedict A: Simulation and matching-based approaches for indirect comparison of treatments. Pharmacoeconomics 2015, 33(6):537-549.

39. Signorovitch J, Erder MH, Xie J, Sikirica V, Lu M, Hodgkins PS, Wu EQ: Comparative effectiveness research using matching-adjusted indirect comparison: an application to treatment with guanfacine extended release or atomoxetine in children with attention-deficit/hyperactivity disorder and comorbid oppositional defiant disorder. Pharmacoepidemiology & Drug Safety 2012, 21 Suppl 2:130-137.

40. Signorovitch JE, Wu EQ, Betts KA, Parikh K, Kantor E, Guo A, Bollu VK, Williams D, Wei LJ, DeAngelo DJ: Comparative efficacy of nilotinib and dasatinib in newly diagnosed chronic myeloid leukemia: a matching-adjusted indirect comparison of randomized trials. Current Medical Research & Opinion 2011, 27(6):1263-1271.

41. Signorovitch JE, Wu EQ, Swallow E, Kantor E, Fan L, Gruenberger JB: Comparative efficacy of vildagliptin and sitagliptin in Japanese patients with type 2 diabetes mellitus: a matching-adjusted indirect comparison of randomized trials. Clinical Drug Investigation 2011, 31(9):665-674.

42. Sikirica V, Findling RL, Signorovitch J, Erder MH, Dammerman R, Hodgkins P, Lu M, Xie J, Wu EQ: Comparative efficacy of guanfacine extended release versus atomoxetine for the treatment of attention-deficit/hyperactivity disorder in children and adolescents: applying matching-adjusted indirect comparison methodology. CNS Drugs 2013, 27(11):943-953.

43. Bergvall N, Rathi H, Nixon RM, Thom HHZ, Alsop J, Dunsire L: Modeling the impact of disease modifying treatment on time to disability health states in multiple sclerosis: An evaluation of oral therapies through indirect comparisons of 6-month confirmed disability progression. Value in Health 2013, 16 (7):A619.

44. Xie J, Juday T, Swallow E, Du X, Uy J, Hebden T, Signorovitch J: Comparative efficacy at 48 weeks of atazanavir/ritonavir versus darunavir/ritonavir in treatment naive HIV-1 patients: A matching adjusted indirect comparison of randomized trials. Value in Health 2012, 15 (4):A10.

45. Xie J, Juday TR, Swallow E, Du X, Uy J, Hebden T, Signorovitch J: Matching-adjusted indirect comparison of lipid profile at 48 weeks among treatment naive HIV-1 patients treated with atazanavir/ritonavir versus darunavir/ritonavir. Value in Health 2013, 16 (3):A79.

46. Donegan S, Williamson P, D'Alessandro U, et al. Assessing key assumptions of network meta-analysis: a review of methods. Research synthesis methods 2013;4(4):291-323.

# References in additional file 1

1. EPOC Group. Modified from the Cochrane EPOC Group checklist. Available at: <http://epoc.cochrane.org/sites/epoc.cochrane.org/files/uploads/datacollectionchecklist.pdf>. Secondary Modified from the Cochrane EPOC Group checklist. Available at: <http://epoc.cochrane.org/sites/epoc.cochrane.org/files/uploads/datacollectionchecklist.pdf>.

2. Del Giovane C, Vacchi L, Mavridis D, et al. Network meta-analysis models to account for variability in treatment definitions: application to dose effects. Statistics in medicine 2013;32(1):25-39.

3. Veroniki AA, Jackson D, Viechtbauer W, et al. Methods to estimate the between-study variance and its uncertainty in meta-analysis. Research synthesis methods 2015.

4. Lambert PC, Sutton AJ, Burton PR, et al. How vague is vague? A simulation study of the impact of the use of vague prior distributions in MCMC using WinBUGS. Stat Med 2005;24(15):2401-28.

5. Veroniki AA, Vasiliadis HS, Higgins JP, et al. Evaluation of inconsistency in networks of interventions. International journal of epidemiology 2013;42(1):332-45.

6. Stewart LA, Clarke M, Rovers M, et al. Preferred Reporting Items for Systematic Review and Meta-Analyses of individual participant data: the PRISMA-IPD Statement. Jama 2015;313(16):1657-65.

7. Hutton B, Salanti G, Caldwell DM, et al. The PRISMA extension statement for reporting of systematic reviews incorporating network meta-analyses of health care interventions: checklist and explanations. Annals of internal medicine 2015;162(11):777-84.

8. Jansen JP, Trikalinos T, Cappelleri JC, et al. Indirect treatment comparison/network meta-analysis study questionnaire to assess relevance and credibility to inform health care decision making: an ISPOR-AMCP-NPC Good Practice Task Force report. Value in health : the journal of the International Society for Pharmacoeconomics and Outcomes Research 2014;17(2):157-73.

9. Tricco AC, Tetzlaff J, Pham B, et al. Non-Cochrane vs. Cochrane reviews were twice as likely to have positive conclusion statements: cross-sectional study. J Clin Epidemiol 2009;62(4):380-86 e1.

10. Moher D, Dulberg CS, Wells GA. Statistical power, sample size, and their reporting in randomized controlled trials. JAMA 1994;272(2):122-4.

11. Nikolakopoulou A, Chaimani A, Veroniki AA, et al. Characteristics of networks of interventions: a description of a database of 186 published networks. PloS one 2014;9(1):e86754.

12. Lee AW. Review of mixed treatment comparisons in published systematic reviews shows marked increase since 2009. J Clin Epidemiol 2014;67(2):138-43.

13. Turner RM, Davey J, Clarke MJ, et al. Predicting the extent of heterogeneity in meta-analysis, using empirical data from the Cochrane Database of Systematic Reviews. International journal of epidemiology 2012;41(3):818-27.

14. Bafeta A, Trinquart L, Seror R, et al. Analysis of the systematic reviews process in reports of network meta-analyses: methodological systematic review. Bmj 2013;347:f3675.

15. Sobieraj DM, Cappelleri JC, Baker WL, et al. Methods used to conduct and report Bayesian mixed treatment comparisons published in the medical literature: a systematic review. BMJ Open 2013;3(7).

16. Chambers JD, Naci H, Wouters OJ, et al. An assessment of the methodological quality of published network meta-analyses: a systematic review. PloS one 2015;10(4):e0121715.

17. Signorovitch J, Ayyagari R, Cheng D, et al. Matching-adjusted indirect comparisons: A simulation study of statistical performance. Value in Health 2013;16 (3):A48.

18. Xie J, Juday T, Swallow E, et al. Comparative efficacy at 48 weeks of atazanavir/ritonavir versus darunavir/ritonavir in treatment naive HIV-1 patients: A matching adjusted indirect comparison of randomized trials. Value in Health 2012;15 (4):A10.

19. Bergvall N, Rathi H, Nixon RM, et al. Modeling the impact of disease modifying treatment on time to disability health states in multiple sclerosis: An evaluation of oral therapies through indirect comparisons of 6-month confirmed disability progression. Value in Health 2013;16 (7):A619.

20. Veroniki AA, Huedo-Medina TB, Fountoulakis KN. Moving from study-level to patient-level data: individual patient network meta-analysis. Network Meta-Analysis: Evidence Synthesis with Mixed Treatment Comparison: NY: Nova Science Publishers, 2014.

21. Jansen JP, Cope S. Network meta-analysis of individual and aggregate level data. Value in Health 2012;15 (4):A159.

22. Ishak KJ, Proskorovsky I, Benedict A. Simulation and matching-based approaches for indirect comparison of treatments. Pharmacoeconomics 2015;33(6):537-49.

23. Caro JJ, Ishak KJ. No head-to-head trial? simulate the missing arms. Pharmacoeconomics 2010;28(10):957-67.

24. Saramago P, Chuang LH, Soares MO. Network meta-analysis of (individual patient) time to event data alongside (aggregate) count data. BMC Medical Research Methodology 2014;14:105.
